# Supplementary figures and images for: Validation of two novel human activity recognition models for typically developing children and children with Cerebral Palsy
Source: PLoS One. 2024 Sep 23;19(9):e0308853. doi: 10.1371/journal.pone.0308853 (PMC11419372; doi:10.1371/journal.pone.0308853)

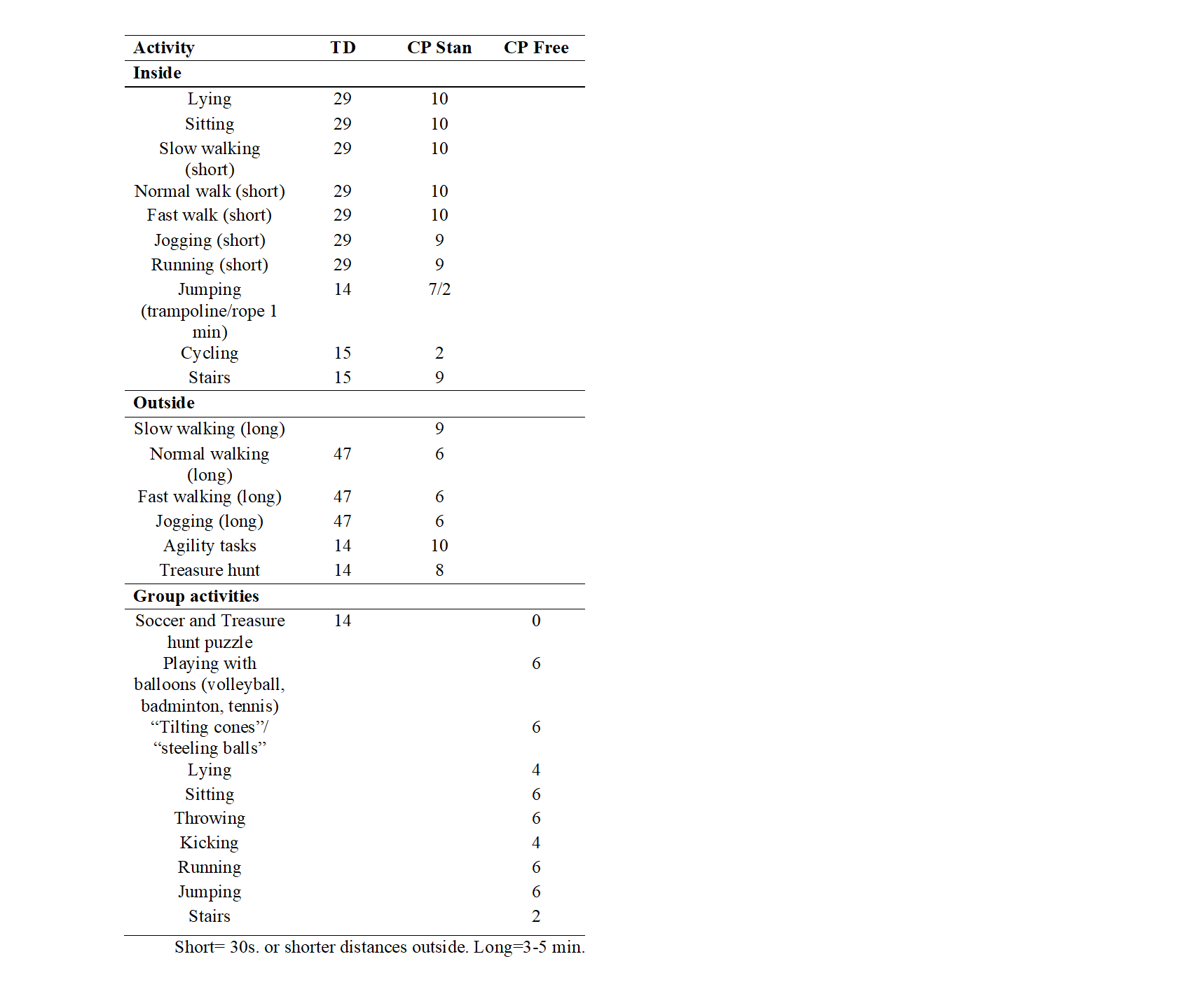

Supplement: S1 Table — (TIF) [file pone.0308853.s001.tif]

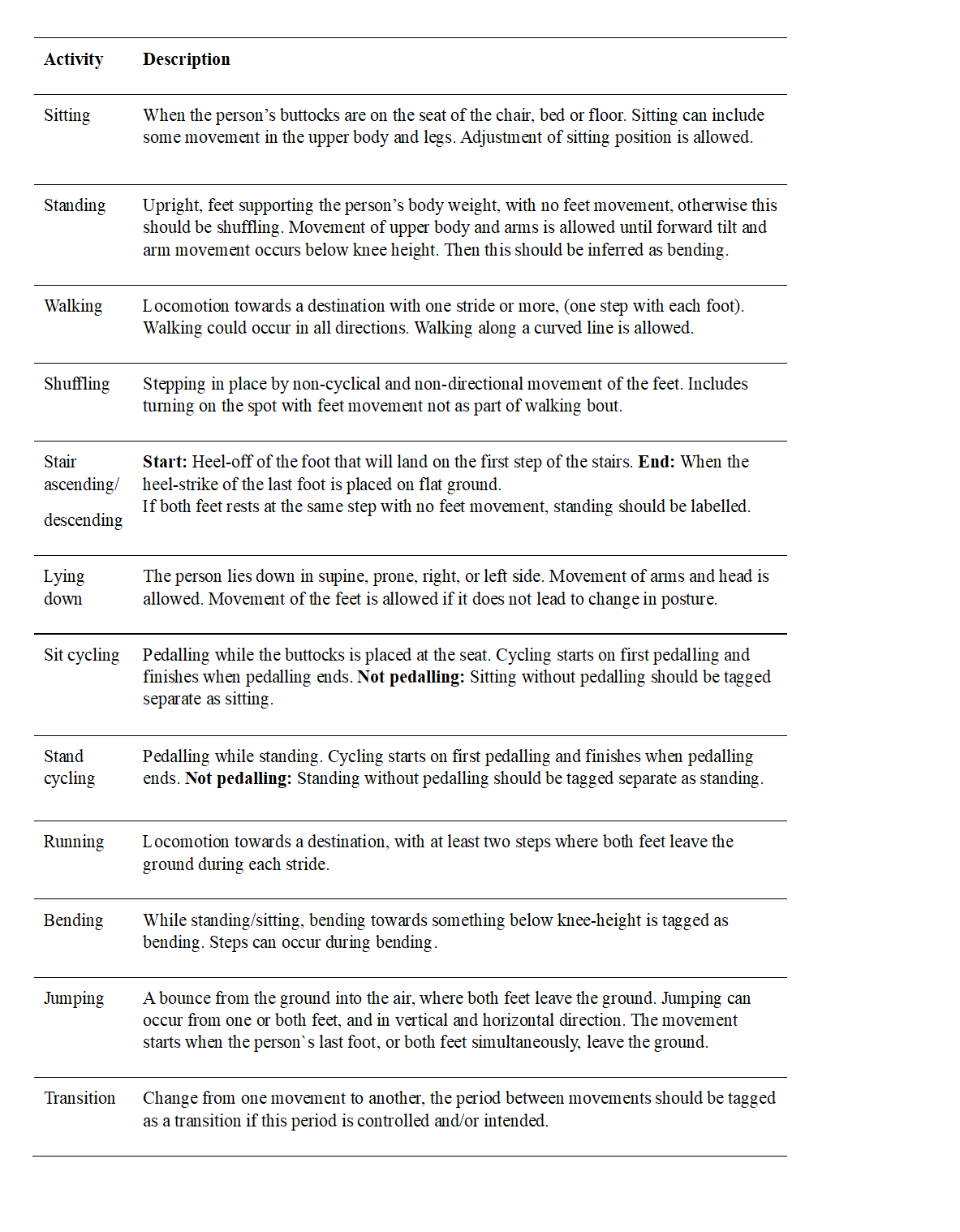

Supplement: S2 Table — (TIF) [file pone.0308853.s002.tif]

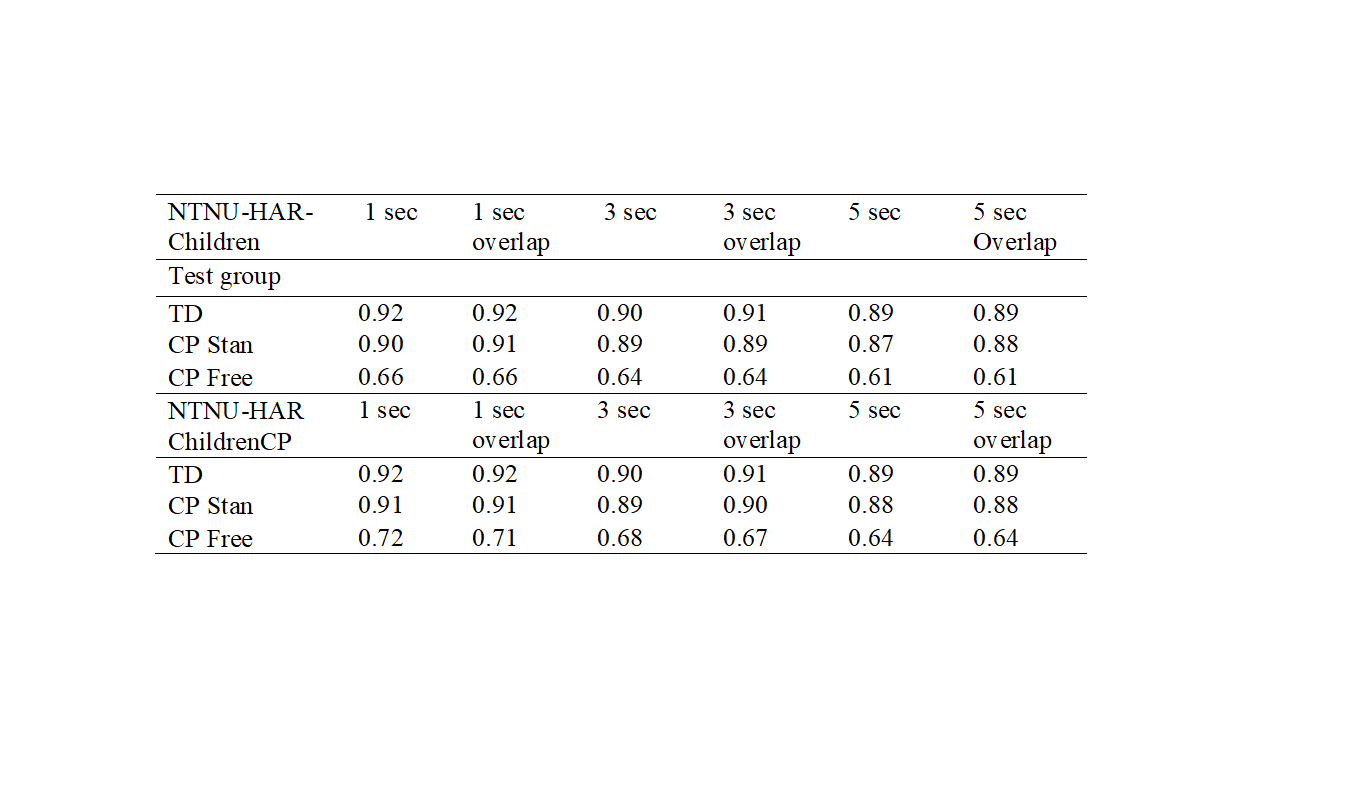

Supplement: S3 Table — Test groups: TD = typically developing children, CP Stan = Cerebral palsy with standardised activities, CP Free = Cerebral palsy with Free play. The range of accuracy is 0–1, where higher scores are better. (TIF) [file pone.0308853.s003.tif]

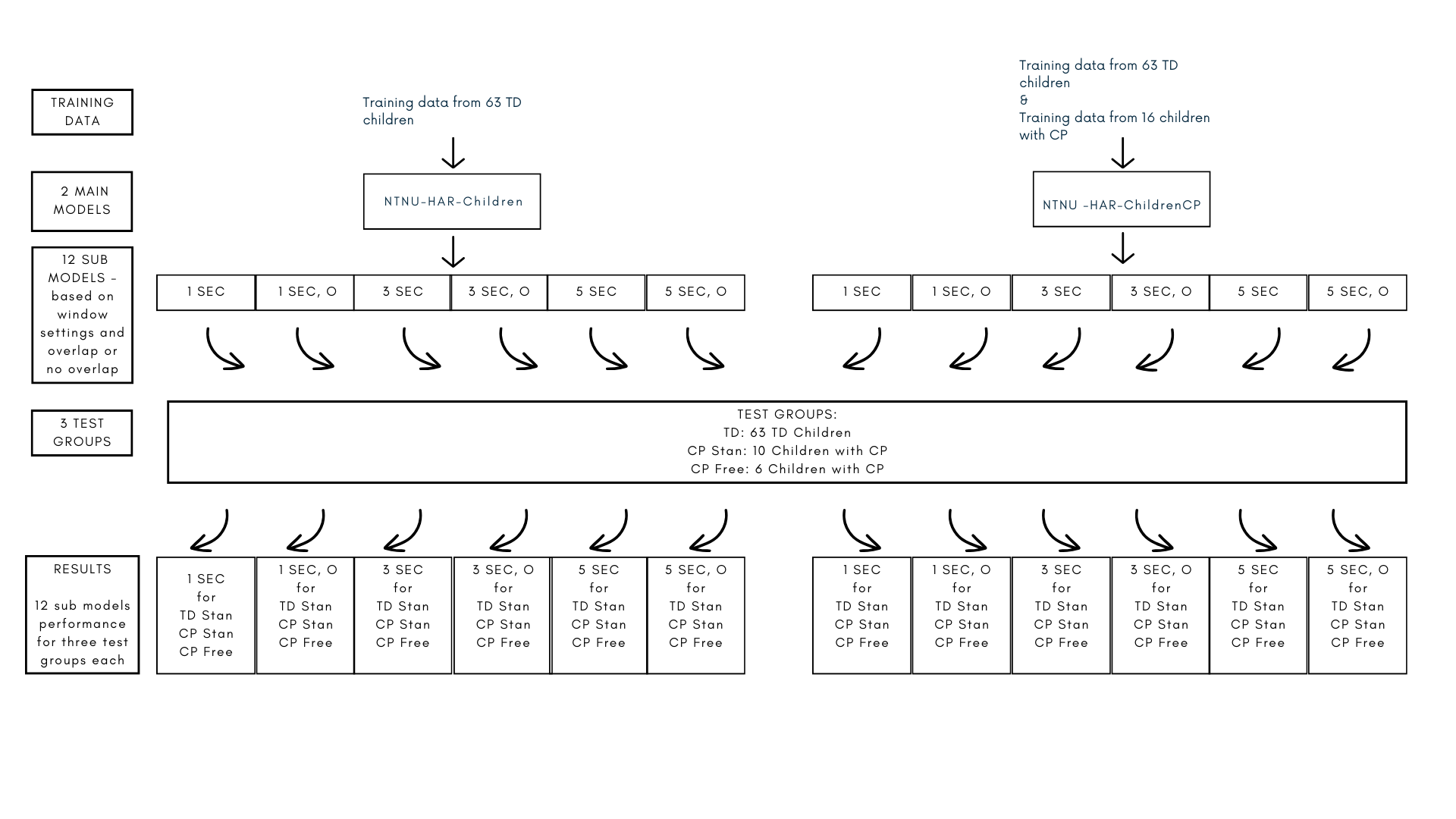

Supplement: S1 Fig — TD children = typically developing children, CP = Cerebral Palsy, O = Overlap, Test groups: TD = typically developing children, CP Stan = Cerebral palsy with standardised activities, CP Free = Cerebral palsy with Free play. (TIF) [file pone.0308853.s004.tif]
