# Supplementary material for: Validation of two novel human activity recognition models for typically developing children and children with Cerebral Palsy
Source: PLoS One. 2024 Sep 23;19(9):e0308853. doi: 10.1371/journal.pone.0308853 (PMC11419372; doi:10.1371/journal.pone.0308853)

PLOS ONE

Validation of two novel human activity recognition models for typically developing children and children with Cerebral Palsy.

--Manuscript Draft--

**Manuscript Number:**	PONE-D-23-37915
**Article Type:**	Research Article
**Full Title:**	Validation of two novel human activity recognition models for typically developing children and children with Cerebral Palsy.
**Short Title:**	Validation of HAR models for children and children with Cerebral Palsy.
**Corresponding Author:**	Marte Fossflaten Tørring, MSc<br>Norwegian University of Science and Technology: Norges teknisk-naturvitenskapelige universitet<br>Trondheim, NORWAY
**Keywords:**	Human Activity Recognition; cerebral palsy; Activity; Children; Machine Learning; Habitual Physical Activity; Free Play; Accelerometer
**Abstract:**	Human Activity Recognition models have potential to contribute to valuable and detailed knowledge of habitual physical activity for typically developing children and children with Cerebral Palsy. The main objective of the present study was to develop and validate two Human Activity Recognition models. One trained on data from typically developing children (n=63), the second also including data from children with Cerebral Palsy (n=16), engaging in standardised activities and free play. Additionally, we aimed to investigate the influence of window settings on model performance. Utilizing the Extreme gradient boost (XGBoost) classifier, twelve sub-models were created, with 1-,3- and 5-seconds windows, with and without overlap. Both Human Activity Recognition models demonstrated excellent predictive capabilities (>92%) for standardised activities for both typically developing and Cerebral Palsy. From all window sizes, the 1-second window performed best for all test groups. Accuracy was slightly lower (>75%) for the Cerebral Palsy test group performing free play activities. The impact of window size and overlap varied depending on activity. In summary both Human Activity Recognition models effectively predict standardised activities, surpassing prior models for typically developing and children with Cerebral Palsy. Notably, the model trained on combined typically developing children and Cerebral Palsy data performed exemplary across all test groups. Researchers should select window settings aligned with their specific research objectives.
**Order of Authors:**	Marte Fossflaten Tørring, MSc
	Aleksej Logacjov, MSc
	Astrid Ustad, MSc
	Siri Merete Brændvik, PhD
	Karin Roeleveld, PhD
	Ellen Marie Bardal, PhD
**Additional Information:**	
**Question**	**Response**
**Financial Disclosure**<br><br>Enter a financial disclosure statement that describes the sources of funding for the work included in this submission. Review the submission guidelines for detailed requirements. View published research articles from *PLOS ONE* for specific	The author(s) received no specific funding for this work.

Powered by Editorial Manager® and ProduXion Manager® from Aries Systems Corporation

examples.<br><br>This statement is required for submission and **will appear in the published article** if the submission is accepted. Please make sure it is accurate.<br><br>**Funded studies**<br>Enter a statement with the following details:<br>• Initials of the authors who received each award<br>• Grant numbers awarded to each author<br>• The full name of each funder<br>• URL of each funder website<br>• Did the sponsors or funders play any role in the study design, data collection and analysis, decision to publish, or preparation of the manuscript?<br><br>Did you receive funding for this work?	
**Competing Interests**<br><br>Use the instructions below to enter a competing interest statement for this submission. On behalf of all authors, disclose any competing interests that could be perceived to bias this work—acknowledging all financial support and any other relevant financial or non-financial competing interests.<br><br>This statement is required for submission and **will appear in the published article** if the submission is accepted. Please make sure it is accurate and that any funding sources listed in your Funding Information later in the submission form are also declared in your Financial Disclosure statement.<br><br>View published research articles from *PLOS ONE* for specific examples.	The authors have declared that no competing interests exist.

**NO authors have competing interests**<br><br>Enter: *The authors have declared that no competing interests exist.*<br><br>**Authors with competing interests**<br><br>Enter competing interest details beginning with this statement:<br><br>*I have read the journal's policy and the authors of this manuscript have the following competing interests: [insert competing interests here]*<br><br>* typeset	
**Ethics Statement**<br><br>Enter an ethics statement for this submission. This statement is required if the study involved:<br><br>• Human participants<br>• Human specimens or tissue<br>• Vertebrate animals or cephalopods<br>• Vertebrate embryos or tissues<br>• Field research<br><br>Write "N/A" if the submission does not require an ethics statement.<br><br>General guidance is provided below. Consult the submission guidelines for detailed instructions. **Make sure that all information entered here is included in the Methods section of the manuscript.**	The study was approved by the Norwegian Centre for Research Data (NSD-nr:50683). In addition, the study was reported to the Regional Ethical Committee for Medical and Health science (REK-nr:2016/707/REK nord) but was not classified under the act on medical and health research.

**Format for specific study types**<br><br>**Human Subject Research (involving human participants and/or tissue)**<br>• Give the name of the institutional review board or ethics committee that approved the study<br>• Include the approval number and/or a statement indicating approval of this research<br>• Indicate the form of consent obtained (written/oral) or the reason that consent was not obtained (e.g. the data were analyzed anonymously)<br><br>**Animal Research (involving vertebrate animals, embryos or tissues)**<br>• Provide the name of the Institutional Animal Care and Use Committee (IACUC) or other relevant ethics board that reviewed the study protocol, and indicate whether they approved this research or granted a formal waiver of ethical approval<br>• Include an approval number if one was obtained<br>• If the study involved *non-human primates*, add *additional details* about animal welfare and steps taken to ameliorate suffering<br>• If anesthesia, euthanasia, or any kind of animal sacrifice is part of the study, include briefly which substances and/or methods were applied<br><br>**Field Research**<br><br>Include the following details if this study involves the collection of plant, animal, or other materials from a natural setting:<br>• Field permit number<br>• Name of the institution or relevant body that granted permission	
**Data Availability**<br><br>Authors are required to make all data underlying the findings described fully available, without restriction, and from the time of publication. PLOS allows rare exceptions to address legal and ethical concerns. See the PLOS Data Policy and FAQ for detailed information.	Yes - all data are fully available without restriction

A Data Availability Statement describing where the data can be found is required at submission. Your answers to this question constitute the Data Availability Statement and **will be published in the article**, if accepted.<br><br>**Important:** Stating 'data available on request from the author' is not sufficient. If your data are only available upon request, select 'No' for the first question and explain your exceptional situation in the text box.<br><br>Do the authors confirm that all data underlying the findings described in their manuscript are fully available without restriction?	
**Describe where the data may be found in full sentences. If you are copying our sample text, replace any instances of XXX with the appropriate details.**<br><br>• If the data are **held or will be held in a public repository**, include URLs, accession numbers or DOIs. If this information will only be available after acceptance, indicate this by ticking the box below. For example: *All XXX files are available from the XXX database (accession number(s) XXX, XXX.*).<br>• If the data are all contained **within the manuscript and/or Supporting Information files**, enter the following: *All relevant data are within the manuscript and its Supporting Information files.*<br>• If neither of these applies but you are able to provide **details of access elsewhere**, with or without limitations, please do so. For example:<br><br>*Data cannot be shared publicly because of [XXX]. Data are available from the XXX Institutional Data Access / Ethics Committee (contact via XXX) for researchers who meet the criteria for access to confidential data.*<br><br>*The data underlying the results presented in the study are available from (include the name of the third party*	All XXX files are available from GitHub database (accession number(s) XXX, XXX.)

and contact information or URL).<br>• This text is appropriate if the data are owned by a third party and authors do not have permission to share the data.<br><br>* typeset	
Additional data availability information:	Tick here if the URLs/accession numbers/DOIs will be available only after acceptance of the manuscript for publication so that we can ensure their inclusion before publication.

Manuscript Click here to access/download;Manuscript;manuscript.docx

Validation of two novel human activity recognition models for typically developing children and children with Cerebral Palsy.

Validation of HAR models for children and children with Cerebral Palsy.

Marte Fossflaten Tørring [1,4*], Aleksej Logacjov [2], Siri Merete Brændvik[1,3], Astrid Ustad [1], Karin Roeleveld [1], Ellen Marie Bardal [1,3].

[1] Department of Neuromedicine and Movement Science, Faculty of Medicine and Health Sciences, Norwegian University of Science and Technology, NTNU, Trondheim, Norway

[2] Department of Computer Science, Faculty of Information Technology and Electrical Engineering, Norwegian University of Science and Technology, NTNU, Trondheim, Norway

[3] Clinic of Rehabilitation, St Olavs Hospital, Trondheim University Hospital, Trondheim, Norway

[4] Physiotherapy Unit, Trondheim Municipal, Trondheim, Norway

*** Correspondence:**

E-mail: *marte.f.torring@ntnu.no* ***(MFT)***

Author contributions

Conceptualization: Ellen Marie Bardal, Siri Merete Brændvik, Karin Roeleveld

Data curation: Marte Fossflaten Tørring, Astrid Ustad, Aleksej Logacjov, Karin Roeleveld, Ellen Marie Bardal

Formal analysis: Marte Fossflaten Tørring, Karin Roeleveld

Funding acquisition: Siri Merete Brændvik, Ellen Marie Bardal, Karin Roeleveld

Methodology: Marte Fossflaten Tørring, Ellen Marie Bardal, Aleksej Logacjov, Astrid Ustad

Project Administration: Siri Merete Brændvik, Ellen Marie Bardal, Karin Roeleveld

Software: Marte Fossflaten Tørring, Aleksej Logacjov

Supervision: Siri Merete Brændvik, Karin Roeleveld

Visualization: Marte Fossflaten Tørring

Writing-original draft: Marte Fossflaten Tørring

Writing- Review and & editing: Marte Fossflaten Tørring, Aleksej Logacjov, Astrid Ustad, Ellen Marie Bardal, Karin Roeleveld, Siri Merete Brændvik

Abstract

Human Activity Recognition models have potential to contribute to valuable and detailed knowledge of habitual physical activity for typically developing children and children with Cerebral Palsy. The main objective of the present study was to develop and validate two Human Activity Recognition models. One trained on data from typically developing children (n=63), the second also including data from children with Cerebral Palsy (n=16), engaging in standardised activities and free play. Additionally, we aimed to investigate the influence of window settings on model performance. Utilizing the Extreme gradient boost (XGBoost) classifier, twelve sub-models were created, with 1-,3- and 5-seconds windows, with and without overlap. Both Human Activity Recognition models demonstrated excellent predictive capabilities (>92%) for standardised activities for both typically developing and Cerebral Palsy. From all window sizes, the 1-second window performed best for all test groups. Accuracy was slightly lower (>75%) for the Cerebral Palsy test group performing free play activities. The impact of window size and overlap varied depending on activity. In summary both Human Activity Recognition models effectively predict standardised activities, surpassing prior models for typically developing and children with Cerebral Palsy. Notably, the model trained on combined typically developing children and Cerebral Palsy data performed exemplary across all test groups. Researchers should select window settings aligned with their specific research objectives.

1. Introduction

Accurate measures of physical activity (PA) are crucial for evidence-based evaluation and regulation of PA in children and adolescents, as well as for assessing the effectiveness of PA interventions. Acceleration-based sensors have gained popularity in recent years as a supplement, and a more reliable alternative to self-reported data in both intervention and population studies (1, 2). Accelerometers provide acceleration in 3D, which can be used to estimate frequency, intensity, mode and duration of activity and sedentary behaviour, offering valuable insights into natural behaviour in children (3-6).

Traditionally, accelerometer data have been analysed using brand specific software with a count-based approach providing information about the intensity of the performed activities (7, 8). Even though several population- and age-specific cut-off values for the different intensities have been developed, the validity is questionable both in typically developing (TP) children and in children with disabilities (9, 10). Cut-off values for children with cerebral palsy (CP) has for example shown to misclassify 30-40% of the performed intensities (11). An alternative approach is to classify unique behavioural patterns by using Human Activity Recognition (HAR) methods, and thereby identify and recognise specific activity types, such as sitting, jumping, or standing (7, 12, 13). HAR models have shown promising results, with over 90% accuracy in free living conditions for various population groups, including TD preschool children (7, 8). However, accuracy varies in studies involving children, ranging from 62-95%, probably depending on factors like window size, activity protocol and algorithm used (14-16).

Despite the advantages with using HAR models, challenges include the need for specific training data, optimal accelerometer placements, and window size considerations (12, 17, 18). The HAR method identifies unique movement patterns, and HAR models trained on healthy adults may not accurately identify physical activity patterns in children, particularly children

with disabilities which often represents with deviating movement patterns (19, 20). To address this, new HAR models must be developed and trained using data collected from children, including children with disabilities. As CP is the most frequent disability among children, it is suitable to include this group for our endeavour. Cerebral Palsy (CP) is a collective term for various neurological impairments resulting from cerebral injuries before the age of two (21-23). The CP condition may include a wide range of physical impairments like reduced walking speed and stride length (24), impaired balance, (25) and secondary musculoskeletal impairments like contractures and skeletal deformities (26), all which may give deviating movement patterns and constraints with regard to daily activity.

The previous studies validating HAR models for TD children are hard to compare, due to use of different classifiers, window sizes, accelerometer placements and activity protocols (14, 16). Moreover, accuracies often drop significantly when transitioning from laboratory-based to free-living conditions [32]. Only three publications have validated HAR models in children with CP, and they present the same issues regarding the model specifications (20, 27, 28). All these three studies achieved good accuracy for sedentary behaviour (over 80%), but variable results for free-living activities, with some as low as 27% accuracy (28). The protocols used in these studies may not adequately represent free-living activities, lacking elements like ballgames, outdoor activities, and free play (20, 27, 28). Therefore, a new HAR model validated on free living data is needed for both TD and CP.

Various human activities unfold over different durations, which the HAR models need to detect. For instance, a single step takes about 500 milliseconds to complete (29). However, walking as an activity involves multiple steps, and thereby extending the time frame beyond a mere 1-second interval. Consequently, the choice of window size is a contentious issue, impacting accuracy for both cut-off and HAR methods, particulary in children, who change activity frequently (19, 30). Some studies suggest increased accuracy with larger window sizes

(e.g., 15 seconds), while others argue for shorter windows (1-2 seconds) (15, 31). Addtionally the use of overlapping windows, which provides more data points, is in some cases favoured for better classification (32).

To meet the challenges described, the present study aimed to develop and validate two machine learning models for recognizing habitual activities in TD children and ambulatory children with CP. Specifically, the two models differentiate with one model trained with only TD data and the second model with both TD and CP data. Additionally, this study investigated the impact of window size and overlapping versus non-overlapping windows on prediction accuracy, and consider practical considerations related to time and storage use.

2. Materials and Methods

2.1 Participants

Data from 63 TD children and 16 children with CP are included in this validation study (Table 1). The TD children were recruited through a local primary and junior high school, and through colleagues and friends. The children with CP were recruited during a habilitation stay or through outpatient clinic at the local hospital. Only children classified with Gross Motor Function Classification System (GMFCS) I and II were included (33). All participants and/or guardian/parents provided written consent, and the data collection started 19.10.2016 and ended 16.08.2019. The study was approved by the Norwegian Centre for Research Data (NSD-nr:50683). In addition, the study was reported to the Regional Ethical Committee for Medical and Health science (REK-nr:2016/707/REK nord) but was not classified under the act on medical and health research.

Table 1. Participant Characteristics

Subjects	TD	CP
**N (B/G)**	63 (35/28)	16 (8/8)
**Age (years)**	10.5 (+/-2.6) [6-15]	11.4 (+/- 2.2) [8-17]
**Height (cm)**	149.1 (+/-15.7) [117-170]	146.7 (+/-10.8) [129-173]
**Weight (kg)**	42.7 (+/13.0) [21-76]	43.2 (+/-11.0) [25-62]
**GMFCS**		
**I**		10
**II**		6

[1] Mean with (+/- SD) and [range]. TD= Typically developing, CP =Cerebral Palsy, N= Number of subjects, B= Boys, G= Girls, cm= Centimetre, kg= Kilogram, GMFCS= Gross motor function classification scale.

2.2 Validation protocols and test groups

The children conducted standardised semi-structured activities including different modes of running, walking, standing, sitting, and lying down, with varying durations. In addition, they performed a free-living protocol including ball games and free play, conducted both indoors and outdoors. For data-synchronisation purposes, the children also performed heel-drops, or the researcher flicked the accelerometer three times. When testing the HAR models, we divided the participants into three groups, based on activity protocol and if they were diagnosed with CP. The TD group included the typically developing children who completed both standardised activities and five minutes of free play. One CP group, here after called CP Stan, are children with CP who completed the same standardised activities as the TD group, except for the five minutes of free play. The second CP group, here after called CP Free, included children with CP who only engaged in group free play activities. For both CP and TD, all activities were performed in a single session, and all TD participants conducted the whole protocol. See Supporting information S1 for full list of activities and number of children conducting the different activities.

2.3 Instrumentation

2.3.1 Activity monitors

All participants wore two Axivity AX3 accelerometers (Axivity Ltd, Newcastle, UK), one on the thigh placed along the anterior midline, in the middle between anterior superior iliac spine and proximal patella, and one at the approximate placement of L3. For the CP group the thigh accelerometers were placed on the least affected side. Acceleration was sampled at 100 Hz and 200 Hz for TD and 100 Hz for CP (range ± 8g).

2.3.2 Video Recordings

Video recording using GoPro Hero 3+ cameras were used to identify the performed activities. The cameras were mounted in corners of the room during inside protocols, play and group activities. For activities with longer duration and/outside activities the GoPro camera was attached with a chest harness, pointing downwards to detect leg movement, or handheld by researcher. The recordings were sampled at 60 frames per second, resolution of 1080x720 pixels.

2.4 Data processing:

2.4.1 Video annotation

The video recordings were used as the ground truth for activity types. The activities in the videos were manually labelled (annotated) frame by frame for each participant using Anvil video annotation tool (version 6) (34). Thirteen activities were labelled using activity definitions used in previous validation studies with the NTNU-HAR models (7, 12, 35). Definitions of activities are listed in Supporting information S2.

2.4.2 Data pre-processing and feature extraction

Before training our machine learning model, we performed three pre-processing steps (Fig 1). Initially we down sampled and synchronized thigh and back accelerometer signals with activity annotations ensuring data alignment at the recommended 50 Hz (13). Subsequently, we segmented the signals into signal frames of our selected window sizes (1 sec, 3 sec, and 5 sec), with and without 50% overlap. Majority voting was applied to the annotations, such that each 1, 3, or 5 second signal frames corresponded to exactly one activity, based on the most frequently occurring in the set window. Lastly, we computed 161 time- and frequency-domain features for each signal frame, using the movement and gravitational components of all six sensor axes, and each sensor's vector magnitude, as described in Logacjov, Bach (12). These resulting features and annotations were used to train the machine learning models.

2.4.3 Machine learning approach

We used the Extreme gradient boost (XGBoost) classifier as our machine learning method. The XGBoost is an ensemble learning approach based on the gradient boosting algorithm (36), where multiple weak classifiers (e.g., decision trees) are trained in a sequential manner. Each weak classifier was trained to minimize the errors made by the previous weak classifier. Our final model prediction is the weighted sum of all weak classifiers' predictions.

Figure 1. The pre-processing and feature extraction, before model training.

2. 4. 4 Model training and cross validation (evaluation)

The model training resulted in two HAR models, NTNU-HAR-Children (HAR-Children) and NTNU-HAR-ChildrenCP (HAR-ChildrenCP). These models comprise a total of 12 sub-models, covering 1-, 3-, and 5-seconds windows with and without overlapping windows. The HAR-Children were trained with data from the TD group and the HAR-ChildrenCP was trained

with additional data from children with CP. For each model we initially performed a 6-fold cross-validation with hyperparameter optimization in the form of a grid search. This allowed us to find optimal hyperparameters for each of the 12 models, leading to a fairer comparison. After finding optimal hyperparameters for each model, we performed 12 leave-one-subject-out cross-validations (LOSOCV), one for each model. In the LOSOCV, the model was trained on all participants, except for one, and this participant became the test-data. The overall performance of the model was estimated by repeating this process for each individual in the dataset and then averaging the performance across each individual (12, 16). This gave us less subject-dependent estimates and thereby less subject-based bias (12, 16, 37). Note that the six HAR-Children models were trained without the CP data. Hence, the performed LOSOCVs only provided test results for the TD group in this model. To get the results for the CP groups from the HAR-Children models we trained the model on the whole TD dataset, and then used the CP data as test data. Additionally, before we compared the results of the different window sizes (1 sec, 3 sec, 5 sec), we unfolded the model predictions to the original 50 samples per second, to make all our models comparable. The complete dataset is available at (link to the dataset available upon paper acceptance) and is named NTNU-Children.

2.5 Data post processing

Initially we annotated with all activity definitions provided in supporting information (S2), we choose to do this to provide precision and to avoid confusing the model when training it on with similar movements. For the further processing, we collapsed some activity labels to make it applicable for practical use. We have defined shuffling as standing with small foot movements (see Supporting information S2), and for our current focus the differentiation between standing still and standing with some foot movement is of limited significance. Therefore, shuffling was imbedded into standing. Similarly, bending is an activity that typically

occur when standing and is therefore collapsed with standing. To avoid confusing our model's ability to recognize level walking, walking up and down stairs was collapsed with walking. The original two categories of cycling, sit cycling and stand cycling were collapsed into cycling, as our primary interest was in recognize the cyclic leg movements. These collapsed activity classes are the same as used in previous NTNU-HAR models (7, 12, 35). The overall preliminary results are provided in Supporting information S3, in their originally annotated form.

2.6 Statistical Analysis

We assessed the HAR models by calculating the overall accuracy for each test group and determining precision, sensitivity, specificity, and F1 Score for each activity type. Sensitivity measured our model's ability to correctly classify activities when they occurred, while specificity evaluated the ability to avoid false recognition when activities were absent. Precision indicated the ratio of correctly classified activities to the sum of correctly and falsely classified activities. The F1 Score, a harmonic mean between precision and sensitivity, provided a weighted precision and sensitivity measure. Accuracy was calculated as the ratio of correctly recognized activity samples to the total number of activity samples. These metrics range from 0-1, with higher values indicating superior performance. The confusion matrixes include the same collapsed activity classes. If the subject did not conduct the activity, they were taken out of the average calculations. We performed all these calculations for each subject before calculating group mean and confidence intervals. All calculations were executed in MATLAB.

3. Results

Fig 2 shows the distribution of the performed activities in the three test groups. There was more data from TD children (total 2997.9 minutes) than CP, and more with CP standardised (total 647.4 minutes) than CP Free (total 180 minutes). In all three test groups there were more time spent with walking, standing, sitting, and running than the other activity labels (Fig 2).

Figure 2. Bar plot of total amount of activity in the three test groups: TD=Typically developing children, CP Stan= Children with CP, standardised activities, CP Free=Children with CP, free play.

3.1 Overall performance of the twelve HAR-models

For all models the overall accuracy was high, with the same median value (0.93) (Table 2), and the accuracy slightly favoured (range: 0.12-0.18) the TD and CP Stan groups compared to CP Free. The 1-second model in both HAR-Children and HAR-ChildrenCP performed exemplary for the respective test groups (Table 2). The largest difference in accuracy was between the corresponding HAR-Children and HAR-ChildrenCP models for the test group CP Free (range: 0.2-0.4, see Table 2). As illustrated in Table 2 the difference between overlap and non-overlap is only present in HAR-Children 1- and 3-seconds, and from this point on we present the non-overlapping models. Non-overlapping models are preferred for their efficiency in processing time and storage, making them more practical for later use. Accuracy for the original activity classes is presented in Supporting information S3. All experiment results can be found at GITHUB (link available after paper acceptance).

Table 2. Overall accuracy for each model with and without overlaps. Test groups: TD= Typically developing children, CP Stan= Cerebral palsy with standardised activities, CP Free= Cerebral palsy with Free play. The range of accuracy is 0-1, where higher scores are better.

NTNU-HAR-Children	1 sec	1 sec overlap	3 sec	3 sec overlap	5 sec	5 sec Overlap
Test group						
TD	0.94	0.95	0.93	0.93	0.92	0.92
CP Stan	0.94	0.94	0.94	0.94	0.93	0.93
CP Free	0.78	0.79	0.77	0.78	0.75	0.75
NTNU-HAR ChildrenCP	1 sec	1 sec overlap	3 sec	3 sec overlap	5 sec	5 sec overlap
TD	0.94	0.94	0.94	0.94	0.92	0.92
CP Stan	0.95	0.95	0.94	0.94	0.93	0.93
CP Free	0.82	0.82	0.81	0.80	0.78	0.77

3.2 Specific activity performance of the six selected HAR-models

In both 1-second models (Table 3a and 3b) all activities were predicted with high accuracy for the TD group, with F1 Score over 0.85. In CP Stan only running (range: 0.79-0.80) and cycling (range: 0.75-0.78) had a F1 Score under 0.90. The CP Free group had slightly lower accuracy and a wider range in both 1-second models (range: 0.29-0.94). This group had superior values in favour of the HAR-Children CP model, with an average difference of 0.09 (Table 3b), where walking, running, and jumping had the largest difference (0.09, 0.07, 0.27).

In the two 3-seconds models (Table 3c and 3d) all activities had still high accuracy for TD and CP Stan (range: 0.78-0.99), both the difference from the corresponding 1-second models (average: 0.01-0.02) and between the 3-seconds models (Table 3c and 3d) were small (F1 Score <0.03). For the CP Free group, the HAR-ChildrenCP 3-seconds (Table 3d) had better F1 Scores (diff: 0.02-0.06) than HAR-Children 3-seconds (Table 3c). However, for all activities the HAR-ChildrenCP 1-second model (Table 3b) were slightly better (range: 0.01-0.04) than 3-seconds (Table 3d).

For the TD group the HAR-Children 5-seconds model (Table 3e) had lower F1 Scores in all activities (range of decrease in F1: 0.01-0.09) compared to 1- and 3-seconds models (Table 3a, b, c, d), except for cycling, where it was higher (< 0.06 increase in F1) with the 5-seconds

models. In the two CP groups the HAR-ChildrenCP 5-seconds (Table 3f) had higher F1 Scores (range increase in F1: 0.01-0.04) than HAR-Children 5-seconds (Table 3e), except for the activity lying. When comparing the HAR-ChildrenCP 1-second (Table 3b) and the 5-seconds model (Table 3f) for the CP Stan group, the average difference in F1 Score was 0.03. Also, here the cycling score was higher with the 5-seconds model. In CP Free, the average difference in F1 Score was 0.05 in favour of the 1-second model. Mark that this was without jumping, while jumping shows the largest difference (Table 3e and 3f).

Table 3. Sensitivity, specificity, precision, and F1 Score calculated as mean across all participants in the test group and [95% confidence interval]. Sub=Subjects, number of participants detected with the activity. Each sub-table represent one of the HAR-models for the three predefined test groups. TD= Typically developing, CP stan= Cerebral Palsy standardised activities, CP Free= Cerebral Palsy, Free play.

**a)**	**NTNU HAR-Children- One second**					
Test group	Activity type					
TD		Sub	Sensitivity	Specificity	Precision	F1 Score
	Walking	62	0.94 [0.92, 0.95]	0.98 [0.97, 0.98]	0.95 [0.94, 0.96]	0.94 [0.93, 0.96]
	Running	62	0.96 [0.94, 0.97]	0.99 [0.99, 0.99]	0.91 [0.87, 0.94]	0.92 [0.90, 0.95]
	Standing	62	0.93 [0.89, 0.96]	0.97 [0.97, 0.98]	0.93 [0.91, 0.95]	0.92 [0.89, 0.95]
	Sitting	62	0.98 [0.97, 0.99]	0.99 [0.97, 1.00]	0.97 [0.94, 0.99]	0.97 [0.95, 0.99]
	Lying	29	0.99 [0.98, 1.00]	1.00 [0.99, 1.00]	0.98 [0.94, 1.01]	0.98 [0.95, 1.00]
	Cycling	21	0.87 [0.78, 0.97]	1.00 [1.00, 1.00]	0.93 [0.87, 0.98]	0.89 [0.81, 0.97]
	Jumping	17	0.87 [0.77, 0.96]	1,00 [1.00, 1.00]	0.92 [0.86, 0.98]	0.87 [0.79, 0.95]
CP Stan		Sub	Sensitivity	Specificity	Precision	F1 Score
	Walking	10	0.89 [0.86, 0.93]	0.97 [0.96, 0.98]	0.92 [0.90, 0.94]	0.90 [0.88, 0.93]
	Running	10	0.77 [0.60, 0.94]	1.00 [0.99, 1.00]	0.88 [0.83, 0.93]	0.80 [0.66, 0.94]
	Standing	10	0.95 [0.93, 0.97]	0.95 [0.93, 0.96]	0.92 [0.89, 0.94]	0.93 [0.91, 0.95]
	Sitting	10	0.99 [0.99, 1.00]	1.00 [0.99, 1.00]	0.99 [0.98, 1.00]	0.99 [0.99, 0.99]
	Lying	10	0.99 [0.98, 1.00]	1.00 [1.00, 1.00]	0.99 [0.98, 1.00]	0.99 [0.98, 1.00]
	Cycling	2	0.62 [-1.37, 2.61]	1.00 [1.00, 1.00]	0.99 [0.92, 1.07]	0.75 [-0.80, 2.30]
	Jumping	7	0.91 [0.85, 0.98]	1.00 [1.00, 1.00]	0.95 [0.91, 0.99]	0.93 [0.88, 0.98]
CP Free		Sub	Sensitivity	Specificity	Precision	F1 Score
	Walking	6	0.49 [0.34, 0.64]	0.92 [0.90, 0.95]	0.65 [0.59, 0.70]	0.55 [0.45, 0.65]
	Running	5	0.60 [0.46, 0.73]	0.99 [0.98, 1.00]	0.52 [0.20, 0.84]	0.53 [0.29, 0.77]

	Standing	6	0.88 [0.84, 0.92]	0.70 [0.59, 0.82]	0.80 [0.75, 0.84]	0.84 [0.81, 0.86]
	Sitting	6	0.87 [0.75, 0.99]	0.99 [0.98, 1.00]	0.93 [0.86, 1.00]	0.90 [0.82, 0.97]
	Lying	6	0.89 [0.77, 1.01]	0.99 [0.95, 1.02]	0.71 [0.29, 1.14]	0.74 [0.38, 1.11]
	Jumping	2	0.19 [-0.03, 0.40]	1.00 [1.00, 1.00]	0.72 [-2.91, 4.34]	0.29 [-0.31, 0.90]

**b)**	**NTNU HAR-ChildrenCP- One second**					
Test group	Activity type					
TD		Sub	Sensitivity	Specificity	Precision	F1 Score
	Walking	62	0.94 [0.92, 0.96]	0.97 [0.97, 0.98]	0.95 [0.94, 0.96]	0.94 [0.93, 0.96]
	Running	62	0.96 [0.95, 0.97]	0.99 [0.99, 0.99]	0.90 [0.87, 0.94]	0.92 [0.90, 0.95]
	Standing	61	0.94 [0.93, 0.95]	0.98 [0.97, 0.98]	0.94 [0.92, 0.95]	0.94 [0.93, 0.95]
	Sitting	62	0.98 [0.97, 0.99]	0.99 [0.96, 1.01]	0.97 [0.94, 0.99]	0.97 [0.95, 0.99]
	Lying	29	0.99 [0.99, 1.00]	1.00 [1.00, 1.00]	0.98 [0.95, 1.01]	0.98 [0.97, 1.00]
	Cycling	21	0.90 [0.82, 0.97]	1.00 [1.00, 1.00]	0.95 [0.92, 0.98]	0.92 [0.86, 0.98]
	Jumping	17	0.83 [0.72, 0.93]	1.00 [1.00, 1.00]	0.91 [0.84, 0.98]	0.86 [0.77, 0.94]
CP Stan		Sub	Sensitivity	Specificity	Precision	F1 Score
	Walking	10	0.91 [0.88, 0.94]	0.97 [0.96, 0.98]	0.91 [0.89, 0.94]	0.91 [0.89, 0.93]
	Running	10	0.76 [0.57, 0.95]	1.00 [0.99, 1.00]	0.87 [0.80, 0.94]	0.79 [0.63, 0.95]
	Standing	10	0.95 [0.93, 0.97]	0.96 [0.94, 0.97]	0.93 [0.91, 0.96]	0.94 [0.92, 0.96]
	Sitting	10	0.99 [0.99, 1.00]	1.00 [1.00, 1.00]	0.99 [0.98, 1.00]	0.99 [0.99, 1.00]
	Lying	10	0.99 [0.98, 1.00]	1.00 [1.00, 1.00]	0.99 [0.98, 1.00]	0.99 [0.98, 1.00]
	Cycling	2	0.66 [-1.59, 2.91]	1.00 [1.00, 1.00]	1.00 [1.00, 1.00]	0.78 [-0.88, 2.44]
	Jumping	7	0.93 [0.86, 1.00]	1.00 [1.00, 1.00]	0.95 [0.92, 0.98]	0.94 [0.89, 0.99]
CP Free		Sub	Sensitivity	Specificity	Precision	F1 Score
	Walking	6	0.61 [0.47, 0.75]	0.92 [0.89, 0.95]	0.69 [0.62, 0.76]	0.64 [0.57, 0.71]
	Running	6	0.56 [0.44, 0.67]	0.99 [0.98, 1.00]	0.45 [0.14, 0.76]	0.46 [0.22, 0.71]
	Standing	6	0.89 [0.85, 0.93]	0.79 [0.68, 0.90]	0.85 [0.81, 0.90]	0.87 [0.86, 0.88]
	Sitting	6	0.92 [0.80, 1.04]	1.00 [0.99, 1.00]	0.96 [0.93, 1.00]	0.94 [0.87, 1.00]
	Lying	6	0.88 [0.78, 0.98]	0.99 [0.95, 1.02]	0.76 [0.38, 1.14]	0.76 [0.45, 1.07]
	Jumping	3	0.43 [-0.14, 1.01]	1.00 [1.00, 1.00]	0.84 [0.15, 1.53]	0.56 [-0.04, 1.16]

**c)**	**NTNU HAR-Children- Three seconds**					
Test group	Activity type					
TD		Sub	Sensitivity	Specificity	Precision	F1 Score
	Walking	62	0.93 [0.92, 0.95]	0.96 [0.96, 0.97]	0.94 [0.92, 0.95]	0.93 [0.92, 0.95]
	Running	62	0.94 [0.93, 0.96]	0.99 [0.98, 0.99]	0.89 [0.85, 0.92]	0.91 [0.88, 0.93]
	Standing	62	0.90 [0.87, 0.93]	0.97 [0.97, 0.98]	0.93 [0.92, 0.95]	0.91 [0.87, 0.94]
	Sitting	62	0.97 [0.96, 0.98]	0.98 [0.97, 1.00]	0.96 [0.94, 0.98]	0.96 [0.94, 0.98]
	Lying	29	0.98 [0.97, 0.99]	0.99 [0.98, 1.00]	0.96 [0.90, 1.01]	0.96 [0.91, 1.00]
	Cycling	21	0.88 [0.79, 0.97]	1.00 [1.00, 1.00]	0.93 [0.87, 0.99]	0.90 [0.82, 0.98]
	Jumping	15	0.92 [0.88, 0.96]	1.00 [1.00, 1.00]	0.91 [0.83, 1.00]	0.90 [0.85, 0.96]
CP Stan		Sub	Sensitivity	Specificity	Precision	F1 Score
	Walking	10	0.90 [0.87, 0.93]	0.97 [0.95, 0.98]	0.90 [0.87, 0.93]	0.90 [0.87, 0.93]
	Running	9	0.78 [0.69, 0.88]	1.00 [0.99, 1.00]	0.90 [0.85, 0.96]	0.83 [0.77, 0.89]
	Standing	10	0.94 [0.91, 0.97]	0.95 [0.94, 0.96]	0.92 [0.89, 0.95]	0.93 [0.90, 0.96]
	Sitting	10	0.99 [0.99, 1.00]	0.99 [0.99, 1.00]	0.98 [0.96, 0.99]	0.98 [0.98, 0.99]
	Lying	10	0.99 [0.98, 1.00]	1.00 [1.00, 1.00]	0.99 [0.98, 1.00]	0.99 [0.98, 1.00]
	Cycling	2	0.64 [0.11, 1.17]	1.00 [1.00, 1.00]	0.99 [0.92, 1.07]	0.78 [0.41, 1.14]
	Jumping	7	0.84 [0.71, 0.97]	1.00 [1.00, 1.00]	0.98 [0.95, 1.00]	0.90 [0.81, 0.98]

CP Free		Sub	Sensitivity	Specificity	Precision	F1 Score
	Walking	6	0.58 [0.40, 0.75]	0.88 [0.84, 0.92]	0.59 [0.54, 0.63]	0.57 [0.48, 0.66]
	Running	5	0.49 [0.19, 0.79]	0.98 [0.96, 1.00]	0.43 [0.11, 0.75]	0.41 [0.15, 0.67]
	Standing	6	0.83 [0.78, 0.89]	0.75 [0.62, 0.88]	0.82 [0.77, 0.87]	0.82 [0.79, 0.86]
	Sitting	6	0.86 [0.75, 0.97]	0.99 [0.98, 1.00]	0.91 [0.82, 1.01]	0.88 [0.81, 0.95]
	Lying	6	0.87 [0.75, 1.00]	0.99 [0.95, 1.02]	0.71 [0.31, 1.11]	0.74 [0.39, 1.09]
	Jumping	0	0.00 [0.00, 0.00]	1.00 [1.00, 1.00]	0.00 [0.00, 0.00]	0.00 [0.00, 0.00]
**d)**	**NTNU HAR-ChildrenCP- Three seconds**					
Test group	Activity type					
TD		Sub	Sensitivity	Specificity	Precision	F1 Score
	Walking	62	0.94 [0.92, 0.95]	0.97 [0.96, 0.97]	0.94 [0.93, 0.95]	0.94 [0.93, 0.95]
	Running	62	0.95 [0.94, 0.96]	0.99 [0.98, 0.99]	0.89 [0.86, 0.93]	0.91 [0.89, 0.94]
	Standing	62	0.90 [0.87, 0.93]	0.97 [0.97, 0.98]	0.93 [0.92, 0.95]	0.91 [0.87, 0.94]
	Sitting	62	0.97 [0.96, 0.98]	0.98 [0.97, 1.00]	0.96 [0.93, 0.98]	0.96 [0.94, 0.98]
	Lying	29	0.98 [0.97, 0.99]	1.00 [1.00, 1.00]	0.97 [0.95, 1.00]	0.97 [0.96, 0.99]
	Cycling	20	0.93 [0.88, 0.97]	1.00 [1.00, 1.00]	0.94 [0.88, 1.00]	0.93 [0.88, 0.98]
	Jumping	14	0.92 [0.88, 0.95]	1.00 [1.00, 1.00]	0.95 [0.93, 0.97]	0.93 [0.91, 0.95]
CP Stan		Sub	Sensitivity	Specificity	Precision	F1 Score
	Walking	10	0.91 [0.89, 0.94]	0.96 [0.95, 0.98]	0.90 [0.86, 0.93]	0.90 [0.88, 0.93]
	Running	9	0.79 [0.67, 0.90]	1.00 [0.99, 1.00]	0.89 [0.82, 0.95]	0.82 [0.76, 0.89]
	Standing	10	0.93 [0.90, 0.96]	0.95 [0.94, 0.97]	0.93 [0.90, 0.96]	0.93 [0.90, 0.96]
	Sitting	10	0.99 [0.99, 0.99]	1.00 [0.99, 1.00]	0.98 [0.97, 0.99]	0.99 [0.98, 0.99]
	Lying	10	0.99 [0.98, 1.00]	1.00 [1.00, 1.00]	0.99 [0.98, 1.00]	0.99 [0.98, 1.00]
	Cycling	2	0.66 [0.56, 0.75]	1.00 [1.00, 1.00]	1.00 [1.00, 1.00]	0.79 [0.72, 0.86]
	Jumping	7	0.86 [0.76, 0.96]	1.00 [1.00, 1.00]	0.96 [0.92, 1.01]	0.91 [0.83, 0.98]
CP Free		Sub	Sensitivity	Specificity	Precision	F1 Score
	Walking	6	0.64 [0.49, 0.79]	0.90 [0.87, 0.93]	0.65 [0.58, 0.71]	0.63 [0.57, 0.69]
	Running	5	0.54 [0.33, 0.74]	0.99 [0.97, 1.00]	0.48 [0.29, 0.68]	0.49 [0.31, 0.68]
	Standing	6	0.86 [0.80, 0.91]	0.79 [0.68, 0.91]	0.85 [0.80, 0.91]	0.85 [0.84, 0.87]
	Sitting	6	0.91 [0.79, 1.03]	1.00 [0.99, 1.00]	0.96 [0.93, 1.00]	0.93 [0.86, 1.00]
	Lying	6	0.84 [0.68, 0.99]	0.99 [0.95, 1.02]	0.72 [0.32, 1.12]	0.72 [0.38, 1.05]
	Jumping	0	0.00 [0.00, 0.00]	1.00 [1.00, 1.00]	0.00 [0.00, 0.00]	0.00 [0.00, 0.00]
**e)**	**NTNU HAR-Children- Five seconds**					
Test group	Activity type					
TD		Sub	Sensitivity	Specificity	Precision	F1 Score
	Walking	62	0.93 [0.91, 0.94]	0.96 [0.95, 0.97]	0.92 [0.91, 0.94]	0.92 [0.91, 0.94]
	Running	62	0.93 [0.91, 0.95]	0.99 [0.98, 0.99]	0.87 [0.83, 0.91]	0.89 [0.86, 0.92]
	Standing	62	0.87 [0.84, 0.91]	0.97 [0.96, 0.98]	0.92 [0.90, 0.93]	0.89 [0.85, 0.92]
	Sitting	62	0.96 [0.95, 0.98]	0.98 [0.97, 1.00]	0.95 [0.92, 0.97]	0.95 [0.93, 0.97]
	Lying	29	0.97 [0.95, 0.98]	0.99 [0.99, 1.00]	0.94 [0.89, 0.99]	0.95 [0.91, 0.99]
	Cycling	21	0.88 [0.80, 0.96]	1.00 [1.00, 1.00]	0.93 [0.88, 0.97]	0.89 [0.82, 0.96]
	Jumping	14	0.92 [0.89, 0.96]	1.00 [1.00, 1.00]	0.94 [0.92, 0.97]	0.93 [0.91, 0.95]
CP Stan		Sub	Sensitivity	Specificity	Precision	F1 Score
	Walking	10	0.89 [0.86, 0.92]	0.96 [0.95, 0.98]	0.89 [0.85, 0.93]	0.89 [0.85, 0.92]
	Running	9	0.78 [0.65, 0.92]	0.99 [0.99, 1.00]	0.84 [0.79, 0.90]	0.80 [0.71, 0.88]
	Standing	10	0.92 [0.88, 0.96]	0.95 [0.93, 0.96]	0.91 [0.87, 0.95]	0.92 [0.88, 0.95]
	Sitting	10	0.99 [0.98, 0.99]	0.99 [0.99, 1.00]	0.97 [0.96, 0.99]	0.98 [0.97, 0.99]

	Lying	10	0.99 [0.98, 1.00]	1.00 [1.00, 1.00]	0.98 [0.96, 0.99]	0.98 [0.97, 0.99]
	Cycling	2	0.59[-0.74, 1.92]	1.00 [1.00, 1.00]	0.99 [0.80, 1.17]	0.73[-0.37, 1.83]
	Jumping	7	0.83 [0.67, 0.98]	1.00 [1.00, 1.00]	0.95 [0.89, 1.00]	0.87 [0.77, 0.97]
CP Free		Sub	Sensitivity	Specificity	Precision	F1 Score
	Walking	6	0.59 [0.42, 0.77]	0.87 [0.82, 0.93]	0.58 [0.52, 0.65]	0.57 [0.48, 0.66]
	Running	5	0.52 [0.24, 0.80]	0.98 [0.96, 0.99]	0.33 [0.12, 0.55]	0.37 [0.19, 0.54]
	Standing	6	0.81 [0.73, 0.89]	0.75 [0.59, 0.90]	0.82 [0.76, 0.88]	0.81 [0.76, 0.85]
	Sitting	6	0.82 [0.69, 0.96]	0.99 [0.98, 1.00]	0.89 [0.75, 1.03]	0.85 [0.74, 0.95]
	Lying	6	0.91 [0.83, 0.99]	0.98 [0.95, 1.02]	0.65 [0.26, 1.05]	0.70 [0.36, 1.04]
	Jumping	0	0.00 [0.00, 0.00]	1.00 [1.00, 1.00]	0.00 [0.00, 0.00]	0.00 [0.00, 0.00]

**f)**	**NTNU HAR-ChildrenCP- Five seconds**					
Test group	Activity type					
TD		Sub	Sensitivity	Specificity	Precision	F1 Score
	Walking	62	0.93 [0.92, 0.95]	0.96 [0.95, 0.97]	0.92 [0.91, 0.94]	0.93 [0.91, 0.94]
	Running	62	0.94 [0.92, 0.96]	0.99 [0.98, 0.99]	0.87 [0.83, 0.90]	0.90 [0.87, 0.92]
	Standing	61	0.89 [0.86, 0.91]	0.97 [0.97, 0.98]	0.92 [0.91, 0.94]	0.90 [0.88, 0.92]
	Sitting	62	0.97 [0.96, 0.98]	0.98 [0.96, 1.00]	0.95 [0.92, 0.97]	0.95 [0.94, 0.97]
	Lying	29	0.98 [0.97, 0.99]	1.00 [0.99, 1.00]	0.95 [0.92, 0.98]	0.96 [0.94, 0.98]
	Cycling	21	0.90 [0.84, 0.97]	1.00 [1.00, 1.00]	0.91 [0.86, 0.96]	0.90 [0.84, 0.96]
	Jumping	14	0.92 [0.88, 0.96]	1.00 [1.00, 1.00]	0.93 [0.90, 0.96]	0.92 [0.90, 0.94]
CP Stan		Sub	Sensitivity	Specificity	Precision	F1 Score
	Walking	10	0.90 [0.88, 0.93]	0.96 [0.94, 0.98]	0.89 [0.84, 0.93]	0.90 [0.86, 0.93]
	Running	9	0.82 [0.70, 0.94]	0.99 [0.99, 1.00]	0.85 [0.80, 0.91]	0.83 [0.75, 0.91]
	Standing	10	0.92 [0.87, 0.97]	0.95 [0.94, 0.96]	0.92 [0.89, 0.95]	0.92 [0.88, 0.96]
	Sitting	10	0.99 [0.98, 1.00]	0.99 [0.99, 1.00]	0.97 [0.96, 0.99]	0.98 [0.98, 0.99]
	Lying	10	0.98 [0.97, 1.00]	1.00 [1.00, 1.00]	0.99 [0.98, 1.00]	0.99 [0.98, 0.99]
	Cycling	2	0.70 [0.63, 0.77]	1.00 [1.00, 1.00]	1.00 [1.00, 1.00]	0.82 [0.78, 0.87]
	Jumping	7	0.84 [0.70, 0.98]	1.00 [1.00, 1.00]	0.95 [0.90, 1.00]	0.88 [0.79, 0.97]
CP Free		Sub	Sensitivity	Specificity	Precision	F1 Score
	Walking	6	0.59 [0.37, 0.80]	0.89 [0.82, 0.95]	0.61 [0.52, 0.71]	0.57 [0.47, 0.67]
	Running	5	0.58 [0.24, 0.92]	0.98 [0.95, 1.00]	0.38 [0.24, 0.53]	0.41 [0.28, 0.54]
	Standing	6	0.83 [0.74, 0.91]	0.78 [0.64, 0.91]	0.84 [0.78, 0.90]	0.83 [0.81, 0.85]
	Sitting	6	0.89 [0.77, 1.01]	0.99 [0.99, 1.00]	0.90 [0.76, 1.05]	0.89 [0.78, 1.00]
	Lying	6	0.83 [0.62, 1.05]	0.99 [0.95, 1.02]	0.73 [0.36, 1.10]	0.72 [0.40, 1.05]
	Jumping	0	0.00 [0.00, 0.00]	1.00 [1.00, 1.00]	0.00 [0.00, 0.00]	0.00 [0.00, 0.00]

3.3. Confusion matrixes

In all the confusion matrixes in Figs 3, 4 and 5 the misclassification was higher in the test group CP Free for all HAR-models, where walking was misclassified as standing on average 37% and standing as walking in 10.8% of the instances. Running was more often misclassified as

walking in the two CP groups, with average 34.2% in the CP Free group and 17 % in CP Stan. Jumping was often misclassified in CP Free, with 100% misclassification in both 3- and 5-seconds models, however for CP Stan jumping was well predicted for all models and higher than the TD group. The difference between HAR-Children and HAR-ChildrenCP decreased with window size (Figs 3, 4, 5). Furthermore, the performance for the HAR-ChildrenCP models decreased with increasing windows while the HAR-Children increase for the CP Free test group, however not for the other test groups (Figs 3, 4, 5).

Figure 3. Confusion matrix for the 1 second models with non-overlapping windows. One matrix for each of the three test-groups. Number of subjects are the same as presented in Table 3. The rows represent the video annotated or labelled activity types. The columns represent the predicted activity types. All numbers are percentages.

Figure 4. Confusion matrix for the 3 second models with non-overlapping windows. One matrix for each of the three test-groups. Number of subjects are the same as presented in Table 3. The rows represent the video annotated or labelled activity types. The columns represent the predicted activity types. All numbers are percentages.

Figure 5. Confusion matrix for the 5 second models with non-overlapping windows. One matrix for each of the three test-groups. Number of subjects are the same as presented in Table 3. The rows represent the video annotated or labelled activity types. The columns represent the predicted activity types. All numbers are percentages.

4. Discussion

This study validates two HAR models' ability to predict habitual physical activities in TD children and children with CP (GMFCS I & II). Both models accurately predict standardised activities, with the best overall accuracy for the CP Stan group. The two 1-second models outperform the other models for all test groups. The HAR-ChildrenCP 1-second had preferable performance for all test groups in all activities except for cycling, where 3- or 5-seconds perform better. In the CP Free group there was more variability between models and wider confidence intervals within activities. There were also larger differences in F1 Scores between HAR-Children and HAR-ChildrenCP models when tested on the CP Free group. The results of the present study show that window size is of importance, and ideal size depends on the target activity. The most challenging activities for the models to correctly predict are running, walking, and jumping for the CP groups.

4.1 The difference in performance between HAR-Children and HAR-ChildrenCP

The goal of activity recognition is to correctly predict daily life activities, particularly in free-living situations. Our overall accuracy for TD children ranges from 92-95%, representing an improvement over prior models designed for the same age group and simulated free living, which report accuracy between 62-86% (14, 15). Notably, studies with higher accuracy exists, however tested on a treadmill (16), limiting their applicability to real life situations. The results of the present study align favourably with previous studies including standardised protocols and children with CP, that have achieved accuracy exceeding 90% (20, 27, 28). Moreover, our study's achievement of accuracies >75% in simulated free-living conditions for children with CP is particularly notable, given the limited existing research with suboptimal accuracy (28). Our model trained with free play activities also performs best for the CP Free group, highlighting the importance of specific training data (19, 20).

The two models consistently demonstrate superior performance when applied to the TD and CP Stan groups, compared to the CP Free group. Intriguingly, in some models, the CP Stan group achieves higher overall and activity-specific performance than the TD group. This implies that the HAR models may not be primarily challenged by the deviating movement patterns in CP. Instead, the presence of unstandardised activities and sporadic transitions within free play emerges as a potential challenge for HAR models. In our data the inferior performance for the two groups that include free play, TD and CP Free, might also be due to the limited training data, with mere five minutes of free play in TD and six participants in CP Free. Nevertheless, free play activities or variability in movement pattens might be beneficial for the model's ability to predict standardised activities, both for TD and children with CP. Which can be exemplified by the superior performance of the HAR-ChildrenCP models for both CP Stan and all activities, except for jumping, in the TD group. This is interesting because one would expect that the heterogeneity within the CP population hinders the model's performance. However, in our experiment, this diversity within the CP population could potentially contribute to improved performance for standardised activities, thereby benefiting both TD and CP Stan. Overall, the HAR-ChildrenCP model includes all 79 children and thereby represent a wide variation of movement patterns, and with the contribution of the CP Free group it offers superior performance in most activities for all test groups.

4.2 Accuracy and misclassification of activities

In regards of misclassification of activities, the two groups with standardised activities had a lower misclassification of jumping, compared to free play. We can theorise that structured, sequential jumping, as seen in these protocols, are easier for the models to recognize compared to sporadic jumping or jumping imbedded in other activities in free play. Conversely, even though all jumping from the TD and CP Stan groups were included in the training, it might

lack the specificity required for the CP Free group. The misclassification of jumping as standing and walking was more pronounced in the TD group than the CP Stan group, and thereby supports the notion that the primary challenge is free play activities.

There were better predictions of walking for the TD group compared to the children with CP. This can be attributed to differences in walking patterns between the groups (24, 25). The asymmetric movement during walking in CP creates a distinct difference in accelerometery from TD children (25). Furthermore, children with CP require longer time during acceleration, and have a steeper increase in acceleration measures related to increase in speed (25), which might affect the recommended choice of window size. In our results the 5-second HAR-Children model perform best for CP Free, indicating that without CP training data, the models with smaller window sizes have difficulties detecting short bouts of walking and running with different accelerometric features.

The misclassification of running as walking in the two CP groups can also be attributed to asymmetry, which is exacerbated with increasing musculoskeletal demands during running (38). The misclassification may also be influenced by the representation of different walking and running paces in our training data. In real life scenarios, children tend to transition intermittently between walking and running paces. Therefore, it is crucial for the model to recognize the activities regardless of pace. Notably, this limitation in differentiating walking paces is consistent with other publications on children with CP, none of which include running (20, 27, 28). Additionally, in existing studies regarding TD children the same misclassifications between walking paces are present, however less misclassification between running and walking (16). The irregularities in gait characteristics among children with CP can also be positive for model performance, where the heterogeneity, might explain the reduced misclassification of walking in both CP groups and TD with the HAR-ChildrenCP model.

4.3. The effect of window size and overlapping windows

Our models confirm the significance of window settings in activity recognition, and its impact depend on the target activity. For instance, momentary activities such as jumping, are in our models best detected with the 1-second window, while cyclic activities with longer cycle lengths such as cycling is better detected with 5-seconds window. Interestingly, when the window size was increased to 5-seconds, the disparity in performance between HAR-Children and HAR-ChildrenCP models diminished in the CP Free group. This suggest that the intermittent changes in the CP Free group are concealed by majority voting in the 5 second window, and conceivably the intermittent activities in the training data play a reduced role for model performance. This data reduction effect of the larger windows have been beneficial in previous research and for specific activities (39), such as walking, cycling, and running, which have cycle rates exceeding one second. Existing literature involving children with CP typically use window sizes ranging from five to fifteen seconds (20, 27, 28, 40), reporting higher F1 Scores with larger windows (20). Ferrari & Micucci (32) advocates for windows that are long enough to capture a complete cycle of a specific movement yet short enough to distinguish between similar movements.

Within the context of evaluating children's daily activities, our data suggest that the 1-second models should be used when the objective is to detect momentary activities, such as jumping. However, it is essential to recognise that these momentary actions often transpire within a broader spectrum of gross motor activities, such as running, walking, and standing, which is often of primary interest. In this context larger window sizes offer a more efficient approach to data reduction and improves model performance in typical daily life scenarios. Hence, in the context of everyday life and health outcomes the utilization of the 3-seconds model emerges as potentially preferrable. This choice is substantiated by our results, where the 3-second model predicts momentary activities, but also activities of extended duration, including cycling, with commendable accuracy.

Regarding the choice between overlapping or non-overlapping windows, our study reveals marginal difference in model performance with slightly better accuracy observed with overlapping windows. The optimal window size represent a trade-off between processing speed and prediction accuracy (18). Consequently, the benefits of overlapping windows must be weighed against the drawbacks, particularly in clinical application. The time-consuming nature of processing overlapping windows is a notable concern. In our model-training the 1-second with overlap required approximately ten times longer processing time than the 5-seconds without overlap. This substantial time difference is consistent with findings of Dehghani & Sarbishei (41), where segmentation with overlapping windows took twice as long, and training took four times as long compared to non-overlapping. Furthermore, in their study, the memory requirements for overlapping windows were nearly nine times greater (41). Given the trade-off in accuracy, questions arise regarding the practical utility of overlapping windows in clinical context, especially when dealing with large data sets.

4.4. Strengths and limitations

Comparing HAR models present considerable challenges due to variations in classifier usage, study populations, activity protocols, accelerometers, accelerometer placements, among other factors. Our study is unique for our utilization of the XGBoost classifier and two accelerometers. The use of two or more accelerometers and our placements have been emphasized as preferred settings (12, 27). The relatively infrequent use of the XGBoost classifier in HAR research contrasts with the prevalence and recommendations in other fields, and have shown strengths, particularly due to its sequential learning (7, 12). Moreover, our study incorporates specific configurations and specifications that have demonstrated excellence in other population groups (7, 12, 35). Additionally, our study is strengthened by the inclusion of playful behaviour and group activities, and thereby simulated habitual activities

for children and children with CP. Furthermore, our data set is relatively large in comparison to other validation studies including children.

Some limitations warrant consideration. We have limited free play training data, potentially impacting prediction accuracy for the CP Free group. Additionally, the absence of a TD Free group prevents a direct examination of the contrasting effects of free play versus the presence of CP on model performance.

4.5 Future perspectives and implications

The present article underscores the specificity of the HAR method, emphasizing the importance of the training data as well as technical specifications for future research and clinical work. One noteworthy consideration is the necessity for a specific HAR model for CP, GMFCS I &II as the disparity between HAR-children and HAR-ChildrenCP appear relatively minor for standardised activities. Conversely, based on our results we can suggest the call for the HAR-ChildrenCP model, or children with disabilities model, is not restricted to children with CP, but also to TD children. Such a model, like our HAR-ChildrenCP, may offer enhanced versatility by greater variation within the training data, facilitating the recognition of a broader spectrum of movement patterns in the general child population. An intriguing avenue for future research involves training our model with activity data collected in children's daily routines, such as in their home, kindergarten, school, and leisure activities. This expansion into real-world scenarios could yield valuable insights and advance the applicability of our model and HAR methods in child populations.

5. Conclusions

Both HAR models demonstrate precise predictions for standardised activities in both TD children and those with CP, and slightly less precise predictions for free play activities, but

still precises and favourable compared to previous models. Among the three groups, the CP Stan has the most accurate predictions, prompting consideration of the influence of impairment versus free play activities. The NTNU HAR-ChildrenCP model with 1-second window exhibits the highest overall accuracy across all three test groups. However, larger window sizes might be recommended for population measurements. The choice of the optimal window size and overlap depends on the target activity.

Acknowledgements

We thank the children and families that participated in this study, and other contributors that assisted with the data collection especially Roar M. Fenne. We also thank Kerstin Bach for the utilization of the NTNU-HAR method.

References

1. Helmerhorst HHJ, Brage S, Warren J, Besson H, Ekelund U. A systematic review of reliability and objective criterion-related validity of physical activity questionnaires. International Journal of Behavioral Nutrition and Physical Activity. 2012;9(1):1-55.
2. Lavelle G, Noorkoiv M, Theis N, Korff T, Kilbride C, Baltzopoulos V, et al. Validity of the international physical activity questionnaire short form (IPAQ-SF) as a measure of physical activity (PA) in young people with cerebral palsy: A cross-sectional study. Physiotherapy. 2020;107:209-15.
3. Trost SG, O'Neil M. Clinical use of objective measures of physical activity. Br J Sports Med. 2014;48(3):178-81.
4. Matthews CE, Hagströmer M, Pober DM, Bowles HR. Best practices for using physical activity monitors in population-based research. Med Sci Sports Exerc. 2012;44(1 Suppl 1):S68.
5. O'Neil ME, Fragala-Pinkham M, Lennon N, George A, Forman J, Trost SG. Reliability and validity of objective measures of physical activity in youth with cerebral palsy who are ambulatory. Phys Ther. 2016;96(1):37-45.
6. Sousa VE, Lopez KD. Towards usable e-Health. Appl Clin Inform. 2017;8(02):470-90.
7. Bach K, Kongsvold A, Bårdstu H, Bardal EM, Kjærnli HS, Herland S, et al. A Machine Learning Classifier for Detection of Physical Activity Types and Postures During Free-Living. Journal for the Measurement of Physical Behaviour. 2022;1(aop):1-8.

8. Ahmadi MN, Trost SG. Device-based measurement of physical activity in pre-schoolers: Comparison of machine learning and cut point methods. PLoS One. 2022;17(4):e0266970.
9. Trost SG, Loprinzi PD, Moore R, Pfeiffer KA. Comparison of accelerometer cut points for predicting activity intensity in youth. Med Sci Sports Exerc. 2011;43(7):1360-8.
10. Lettink A, Altenburg TM, Arts J, van Hees VT, Chinapaw MJ. Systematic review of accelerometer-based methods for 24-h physical behavior assessment in young children (0–5 years old). International Journal of Behavioral Nutrition and Physical Activity. 2022;19(1):1-63.
11. Trost SG, Fragala-Pinkham M, Lennon N, O'Neil ME. Decision trees for detection of activity intensity in youth with cerebral palsy. Med Sci Sports Exerc. 2016;48(5):958.
12. Logacjov A, Bach K, Kongsvold A, Bårdstu HB, Mork PJ. HARTH: A Human Activity Recognition Dataset for Machine Learning. Sensors. 2021;21(23):7853.
13. Farrahi V, Niemelä M, Kangas M, Korpelainen R, Jämsä T. Calibration and validation of accelerometer-based activity monitors: A systematic review of machine-learning approaches. Gait Posture. 2019;68:285-99.
14. Hagenbuchner M, Cliff DP, Trost SG, Van Tuc N, Peoples GE. Prediction of activity type in preschool children using machine learning techniques. J Sci Med Sport. 2015;18(4):426-31.
15. Ahmadi MN, Pavey TG, Trost SG. Machine learning models for classifying physical activity in free-living preschool children. Sensors. 2020;20(16):4364.
16. Stewart T, Narayanan A, Hedayatrad L, Neville J, Mackay L, Duncan S. A Dual-Accelerometer System for Classifying Physical Activity in Children and Adults. Med Sci Sports Exerc. 2018;50(12):2595-602.
17. Aadland E, Andersen LB, Anderssen SA, Resaland GK, Kvalheim OM. Accelerometer epoch setting is decisive for associations between physical activity and metabolic health in children. J Sports Sci. 2020;38(3):256-63.
18. Banos O, Galvez J-M, Damas M, Pomares H, Rojas I. Window size impact in human activity recognition. Sensors. 2014;14(4):6474-99.
19. Skovdahl P, Olofsson CK, Arvidsson D. Physical activity in children and adolescents with CHD: review from a measurement methodological perspective. Cardiol Young. 2021;31(4):518-31.
20. Ahmadi M, O'Neil M, Fragala-Pinkham M, Lennon N, Trost S. Machine learning algorithms for activity recognition in ambulant children and adolescents with cerebral palsy. J Neuroeng Rehabil. 2018;15(1):1-9.
21. Andersen GL, Irgens LM, Haagaas I, Skranes JS, Meberg AE, Vik T. Cerebral palsy in Norway: prevalence, subtypes and severity. Eur J Paediatr Neurol. 2008;12(1):4-13.
22. Smith B. L Andrew Koman, Beth Paterson Smith, Jeffrey S Shilt. THE LANCET. 2004;363:1619-31.
23. Omura J, Fuentes M, Bjornson K. Participation in daily life: influence on quality of life in ambulatory children with cerebral palsy. PM&R. 2018;10(11):1185-91.
24. Dallmeijer AJ, Brehm M-A. Physical strain of comfortable walking in children with mild cerebral palsy. Disabil Rehabil. 2011;33(15-16):1351-7.
25. Saether R, Helbostad JL, Adde L, Brændvik S, Lydersen S, Vik T. Gait characteristics in children and adolescents with cerebral palsy assessed with a trunk-worn accelerometer. Res Dev Disabil. 2014;35(7):1773-81.
26. Graham HK, Selber P. Musculoskeletal aspects of cerebral palsy. The Journal of bone and joint surgery British volume. 2003;85(2):157-66.
27. Goodlich BI, Armstrong EL, Horan SA, Baque E, Carty CP, Ahmadi MN, et al. Machine learning to quantify habitual physical activity in children with cerebral palsy. Developmental Medicine & Child Neurology. 2020;62(9):1054-60.
28. Ahmadi MN, O'Neil ME, Baque E, Boyd RN, Trost SG. Machine learning to quantify physical activity in children with cerebral palsy: Comparison of group, group-personalized, and fully-personalized activity classification models. Sensors. 2020;20(14):39-76.

29. Thevenon A, Gabrielli F, Lepvrier J, Faupin A, Allart E, Tiffreau V, et al. Collection of normative data for spatial and temporal gait parameters in a sample of French children aged between 6 and 12. Ann Phys Rehabil Med. 2015;58(3):139-44.
30. Migueles JH, Cadenas-Sanchez C, Ekelund U, Delisle Nyström C, Mora-Gonzalez J, Löf M, et al. Accelerometer data collection and processing criteria to assess physical activity and other outcomes: a systematic review and practical considerations. Sports Med. 2017;47:1821-45.
31. Narayanan A, Stewart T, Mackay L. A Dual-Accelerometer System for Detecting Human Movement in a Free-living Environment. Med Sci Sports Exerc. 2020;52(1):252-8.
32. Ferrari A, Micucci D, Mobilio M, Napoletano P. Trends in human activity recognition using smartphones. Journal of Reliable Intelligent Environments. 2021;7(3):189-213.
33. Palisano R, Rosenbaum P, Walter S, Russell D, Wood E, Galuppi B. Development and reliability of a system to classify gross motor function in children with cerebral palsy. Dev Med Child Neurol. 1997;39(4):214-23.
34. Kipp M. ANVIL: A Universal Video Research Tool. . In: Durands J, Gut, U., Kristofferson, G., , editor. Handbook of Corpus Phonology Oxford, UK: Oxford University Press 2014. p. 420-36.
35. Ustad A, Logacjov A, Trollebø SØ, Thingstad P, Vereijken B, Bach K, et al. Validation of an Activity Type Recognition Model Classifying Daily Physical Behavior in Older Adults: The HAR70+ Model. Sensors. 2023;23(5):2368.
36. Friedman JH. Greedy function approximation: a gradient boosting machine. Annals of statistics. 2001:1189-232.
37. Gholamiangonabadi D, Kiselov N, Grolinger K. Deep neural networks for human activity recognition with wearable sensors: Leave-one-subject-out cross-validation for model selection. Ieee Access. 2020;8:133982-94.
38. Böhm H, Döderlein L. Gait asymmetries in children with cerebral palsy: do they deteriorate with running? Gait Posture. 2012;35(2):322-7.
39. Aadland E, Andersen LB, Anderssen SA, Resaland GK, Kvalheim OM. Associations of volumes and patterns of physical activity with metabolic health in children: A multivariate pattern analysis approach. Prev Med. 2018;115:12-8.
40. Rast FM, Jucker F, Labruyere R. Accuracy of sensor-based classification of clinically relevant motor activities in daily life of children with mobility impairments. medRxiv. 2022.
41. Dehghani A, Sarbishei O, Glatard T, Shihab E. A quantitative comparison of overlapping and non-overlapping sliding windows for human activity recognition using inertial sensors. Sensors. 2019;19(22):5026.

Supporting information

S1 Table. Overview of conducted activities for each test group.

S2 Table. Definition of activities.

S3 Table. Overall accuracy for each model with and without overlap and original activity classes. Test groups: TD= typically developing children, CP Stan= Cerebral palsy with standardised activities, CP Free= Cerebral palsy with Free play. The range of accuracy is 0-1, where higher scores are better.

Bar plot of total amount of activity in the three test groups

Click here to access/download;Figure;Figure2.tif

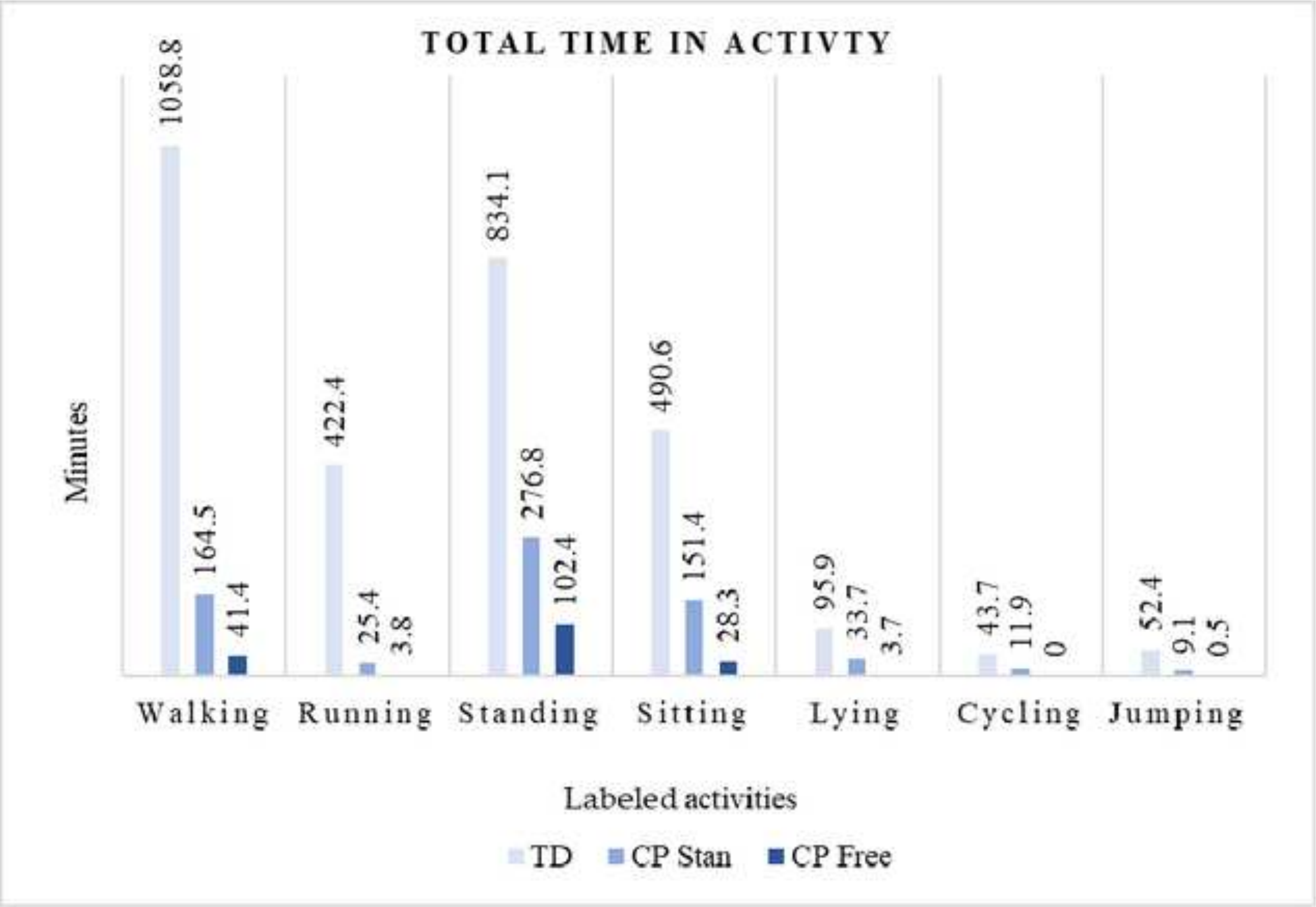

Confusion matrix for the 1 second models

Click here to access/download;Figure;Figure3.tif

NTNU HAR-Children-One second

a) Typical developing children

Labeled \ Predicted	Walking	Running	Standing	Sitting	Lying	Cycling	Jumping
Walking	93.8	1.7	4.0	0.4	0.1	0.0	0.1
Running	2.5	95.7	1.5	0.1	0.1	0.0	0.5
Standing	4.4	0.6	92.7	2.2	0.2	0.1	0.1
Sitting	0.3	0.0	1.4	97.9	0.6	0.3	0.0
Lying	0.1	0.0	0.3	0.7	98.9	0.0	0.0
Cycling	2.6	0.2	6.6	7.5	0.0	83.1	0.0
Jumping	14.1	7.3	13.1	4.2	0.0	0.0	61.3

b) CP- Standardised activities

Labeled \ Predicted	Walking	Running	Standing	Sitting	Lying	Cycling	Jumping
Walking	89.2	0.8	9.8	0.1	0.0	0.0	0.1
Running	14.8	76.8	8.4	0.0	0.0	0.0	0.1
Standing	3.8	0.3	95.2	0.3	0.3	0.1	0.1
Sitting	0.1	0.0	0.6	99.3	0.0	0.0	0.0
Lying	0.0	0.0	0.2	0.7	99.2	0.0	0.0
Cycling	2.9	0.1	33.2	2.0	0.0	61.7	0.0
Jumping	2.1	3.7	3.0	0.0	0.0	0.0	91.1

c) CP- Free play

Labeled \ Predicted	Walking	Running	Standing	Sitting	Lying	Cycling	Jumping
Walking	49.1	3.8	46.8	0.2	0.0		0.2
Running	30.4	49.9	18.5	0.0	0.0		1.4
Standing	10.0	0.5	88.2	1.3	0.0		0.0
Sitting	0.2	0.0	7.4	87.1	5.3		0.0
Lying	0.1	0.0	1.1	9.6	89.1		0.0
Cycling							
Jumping	16.1	7.4	67.1	0.0	0.0		9.4

NTNU HAR-ChildrenCP-One second

a) Typical developing children

Labeled \ Predicted	Walking	Running	Standing	Sitting	Lying	Cycling	Jumping
Walking	93.9	1.8	3.6	0.7	0.0	0.0	0.1
Running	2.3	96.0	1.2	0.1	0.0	0.0	0.6
Standing	4.7	0.7	92.3	2.2	0.1	0.1	0.1
Sitting	0.3	0.0	1.3	97.9	0.6	0.3	0.0
Lying	0.1	0.0	0.3	0.4	99.2	0.0	0.0
Cycling	1.9	0.2	4.4	8.1	0.0	85.5	0.0
Jumping	15.5	10.2	11.6	4.2	0.0	0.0	58.5

b) CP- Standardised activities

Labeled \ Predicted	Walking	Running	Standing	Sitting	Lying	Cycling	Jumping
Walking	91.1	0.8	7.9	0.1	0.0	0.0	0.1
Running	18.7	76.2	5.0	0.0	0.0	0.0	0.1
Standing	4.2	0.3	94.9	0.2	0.3	0.1	0.2
Sitting	0.1	0.0	0.4	99.4	0.0	0.0	0.0
Lying	0.1	0.0	0.1	0.7	99.2	0.0	0.0
Cycling	2.8	0.1	28.9	2.5	0.0	65.7	0.0
Jumping	1.2	2.8	3.1	0.0	0.0	0.0	93.0

c) CP- Free play

Labeled \ Predicted	Walking	Running	Standing	Sitting	Lying	Cycling	Jumping
Walking	61.2	3.5	35.0	0.1	0.0		0.1
Running	25.7	55.6	18.6	0.0	0.0		0.1
Standing	10.3	0.5	88.9	0.2	0.0		0.0
Sitting	0.2	0.0	2.8	91.8	5.2		0.0
Lying	0.1	0.0	0.3	11.8	87.8		0.0
Cycling							
Jumping	16.6	4.2	46.8	0.0	0.0		32.4

Click here to access/download;Figure;Figure4.tif

NTNU HAR-Children- Three seconds

a) Typically developing children

Labeled \ Predicted	Walking	Running	Standing	Sitting	Lying	Cycling	Jumping
Walking	93.3	2.0	3.7	0.6	0.6	0.0	0.0
Running	2.7	94.5	2.3	0.2	0.4	0.1	0.4
Standing	5.9	1.2	90.0	2.4	0.8	0.1	0.2
Sitting	0.9	0.1	1.5	97.1	0.7	0.4	0.0
Lying	0.5	0.0	0.8	0.9	97.8	0.0	0.0
Cycling	6.9	0.0	2.3	7.0	0.0	83.8	0.0
Jumping	17.8	1.8	17.9	5.0	0.0	0.0	57.4

a) CP- Standardised activities

Labeled \ Predicted	Walking	Running	Standing	Sitting	Lying	Cycling	Jumping
Walking	90.0	0.4	9.2	0.3	0.0	0.0	0.1
Running	20.1	70.4	9.5	0.0	0.0	0.0	0.1
Standing	4.9	0.4	93.9	0.5	0.3	0.1	0.0
Sitting	0.3	0.0	0.4	99.2	0.1	0.0	0.0
Lying	0.1	0.0	0.2	1.0	98.7	0.0	0.0
Cycling	10.4	0.0	17.5	8.2	0.0	63.8	0.0
Jumping	2.7	6.5	6.7	0.0	0.0	0.0	84.2

a) CP- Free play

Labeled \ Predicted	Walking	Running	Standing	Sitting	Lying	Cycling	Jumping
Walking	57.9	4.6	37.3	0.2	0.1		0.0
Running	42.8	40.7	16.2	0.4	0.0		0.0
Standing	14.8	0.9	83.1	1.2	0.0		0.0
Sitting	1.6	0.0	7.2	85.8	5.3		0.0
Lying	0.7	0.0	2.4	9.6	87.3		0.0
Cycling							
Jumping	19.4	0.0	80.6	0.0	0.0		0.0

NTNU HAR-ChildrenCP- Three seconds

a) Typical developing children

Labeled \ Predicted	Walking	Running	Standing	Sitting	Lying	Cycling	Jumping
Walking	93.8	1.7	3.7	0.7	0.1	0.1	0.1
Running	2.2	95.1	2.3	0.2	0.0	0.3	0.4
Standing	6.2	1.2	90.0	2.4	0.2	0.1	0.2
Sitting	0.8	0.1	1.4	97.2	0.6	0.6	0.0
Lying	0.5	0.0	0.8	0.9	97.8	0.0	0.0
Cycling	4.0	0.0	2.2	9.6	0.0	84.1	0.0
Jumping	13.1	1.5	27.1	5.0	0.0	0.0	53.4

a) CP- Standardised activities

Labeled \ Predicted	Walking	Running	Standing	Sitting	Lying	Cycling	Jumping
Walking	91.4	0.5	7.7	0.3	0.0	0.0	0.1
Running	18.6	70.8	10.6	0.0	0.0	0.0	0.1
Standing	5.5	0.5	93.4	0.4	0.2	0.0	0.1
Sitting	0.4	0.0	0.5	99.1	0.1	0.0	0.0
Lying	0.2	0.0	0.2	0.8	98.9	0.0	0.0
Cycling	8.0	0.0	21.1	5.3	0.0	65.5	0.0
Jumping	2.8	5.3	5.7	0.0	0.0	0.0	86.1

a) CP- Free play

Labeled \ Predicted	Walking	Running	Standing	Sitting	Lying	Cycling	Jumping
Walking	63.9	3.2	32.8	0.2	0.0		0.0
Running	35.6	44.7	19.3	0.4	0.0		0.0
Standing	13.2	0.9	85.6	0.2	0.0		0.0
Sitting	0.8	0.0	2.9	90.9	5.3		0.0
Lying	0.6	0.0	1.6	14.2	83.7		0.0
Cycling							
Jumping	28.1	4.1	67.8	0.0	0.0		0.0

Confusion matrix for the 5 second models

Click here to access/download;Figure;Figure5.tif

NTNU HAR-Children- Five seconds

a) Typical developing children

Labeled \ Predicted	Walking	Running	Standing	Sitting	Lying	Cycling	Jumping
Walking	92.6	2.0	4.2	0.9	0.5	0.0	0.1
Running	2.7	93.4	3.2	0.5	0.3	0.0	0.2
Standing	7.8	1.7	87.2	2.7	0.9	0.1	0.3
Sitting	1.1	0.0	1.8	96.3	0.8	0.8	0.0
Lying	0.5	0.0	0.9	1.7	97.0	0.0	0.0
Cycling	7.3	0.0	1.8	5.7	0.0	76.8	0.0
Jumping	10.1	1.2	26.5	8.3	0.0	0.0	53.9

a) CP- Standardised activities

Labeled \ Predicted	Walking	Running	Standing	Sitting	Lying	Cycling	Jumping
Walking	88.7	0.7	9.7	0.7	0.1	0.0	0.2
Running	16.8	70.5	12.6	0.1	0.0	0.0	0.1
Standing	6.0	0.7	92.4	0.5	0.3	0.2	0.1
Sitting	0.4	0.0	0.6	98.7	0.3	0.0	0.0
Lying	0.0	0.0	0.3	0.9	98.8	0.0	0.0
Cycling	13.7	0.0	10.4	3.3	0.0	39.2	0.0
Jumping	1.1	6.9	8.9	0.3	0.0	0.0	82.8

a) CP- Free play

Labeled \ Predicted	Walking	Running	Standing	Sitting	Lying	Cycling	Jumping
Walking	59.3	4.8	35.4	0.5	0.1	0.0	0.0
Running	40.3	43.2	15.3	1.3	0.0	0.0	0.0
Standing	16.5	1.8	80.5	1.1	0.1	0.0	0.0
Sitting	1.9	0.1	9.1	82.5	6.1	1.9	0.0
Lying	2.3	0.0	1.7	5.1	90.8	0.0	0.0
Cycling	0.0	0.0	0.0	0.0	0.0	0.0	0.0
Jumping	1.0	0.0	99.0	0.0	0.0	0.0	0.0

NTNU HAR-ChildrenCP- Five seconds

a) Typical developing children

Labeled \ Predicted	Walking	Running	Standing	Sitting	Lying	Cycling	Jumping
Walking	93.1	1.8	4.0	1.0	0.2	0.0	0.1
Running	2.3	94.0	3.1	0.4	0.0	0.0	0.5
Standing	8.0	1.8	87.1	2.7	0.5	0.1	0.4
Sitting	1.2	0.1	1.3	96.7	0.8	0.9	0.0
Lying	0.6	0.0	0.7	0.9	97.8	0.0	0.0
Cycling	6.0	0.0	1.5	6.3	0.0	86.2	0.0
Jumping	14.5	1.1	22.3	8.3	0.0	0.0	53.7

a) CP- Standardised activities

Labeled \ Predicted	Walking	Running	Standing	Sitting	Lying	Cycling	Jumping
Walking	90.2	0.6	8.3	0.7	0.0	0.0	0.1
Running	14.1	74.0	11.7	0.1	0.0	0.0	0.1
Standing	6.9	0.7	91.7	0.5	0.1	0.0	0.1
Sitting	0.4	0.0	0.5	99.0	0.1	0.0	0.0
Lying	0.0	0.0	0.3	1.2	98.5	0.0	0.0
Cycling	6.4	0.0	19.2	4.3	0.0	70.1	0.0
Jumping	1.4	6.3	8.4	0.3	0.0	0.0	83.7

a) CP- Free play

Labeled \ Predicted	Walking	Running	Standing	Sitting	Lying	Cycling	Jumping
Walking	58.5	5.0	35.7	0.6	0.1		0.0
Running	30.7	48.2	19.9	1.3	0.0		0.0
Standing	15.0	1.8	82.6	0.4	0.1		0.0
Sitting	1.2	0.1	4.4	88.8	5.5		0.0
Lying	1.8	0.0	0.8	14.2	83.2		0.0
Cycling							
Jumping	27.7	0.0	72.3	0.0	0.0		0.0

 Click here to access/download;Figure;S1.tif

Activity	TD	CP Stan	CP Free
**Inside**			
Lying	29	10	
Sitting	29	10	
Slow walking (short)	29	10	
Normal walk (short)	29	10	
Fast walk (short)	29	10	
Jogging (short)	29	9	
Running (short)	29	9	
Jumping (trampoline/rope 1 min)	14	7/2	
Cycling	15	2	
Stairs	15	9	
**Outside**			
Slow walking (long)		9	
Normal walking (long)	47	6	
Fast walking (long)	47	6	
Jogging (long)	47	6	
Agility tasks	14	10	
Treasure hunt	14	8	
**Group activities**			
Soccer and Treasure hunt puzzle	14		0
Playing with balloons (volleyball, badminton, tennis)			6
"Tilting cones"/ "steeling balls"			6
Lying			4
Sitting			6
Throwing			6
Kicking			4
Running			6
Jumping			6
Stairs			2

Short= 30s. or shorter distances outside. Long=3-5 min.

 Click here to access/download;Figure;S2.tif

Activity	Description
Sitting	When the person's buttocks are on the seat of the chair, bed or floor. Sitting can include some movement in the upper body and legs. Adjustment of sitting position is allowed.
Standing	Upright, feet supporting the person's body weight, with no feet movement, otherwise this should be shuffling. Movement of upper body and arms is allowed until forward tilt and arm movement occurs below knee height. Then this should be inferred as bending.
Walking	Locomotion towards a destination with one stride or more, (one step with each foot). Walking could occur in all directions. Walking along a curved line is allowed.
Shuffling	Stepping in place by non-cyclical and non-directional movement of the feet. Includes turning on the spot with feet movement not as part of walking bout.
Stair ascending/ descending	**Start:** Heel-off of the foot that will land on the first step of the stairs. **End:** When the heel-strike of the last foot is placed on flat ground.<br>If both feet rests at the same step with no feet movement, standing should be labelled.
Lying down	The person lies down in supine, prone, right, or left side. Movement of arms and head is allowed. Movement of the feet is allowed if it does not lead to change in posture.
Sit cycling	Pedalling while the buttocks is placed at the seat. Cycling starts on first pedalling and finishes when pedalling ends. **Not pedalling:** Sitting without pedalling should be tagged separate as sitting.
Stand cycling	Pedalling while standing. Cycling starts on first pedalling and finishes when pedalling ends. **Not pedalling:** Standing without pedalling should be tagged separate as standing.
Running	Locomotion towards a destination, with at least two steps where both feet leave the ground during each stride.
Bending	While standing/sitting, bending towards something below knee-height is tagged as bending. Steps can occur during bending.
Jumping	A bounce from the ground into the air, where both feet leave the ground. Jumping can occur from one or both feet, and in vertical and horizontal direction. The movement starts when the person's last foot, or both feet simultaneously, leave the ground.
Transition	Change from one movement to another, the period between movements should be tagged as a transition if this period is controlled and/or intended.

 Click here to access/download;Figure;S3.tif

NTNU-HAR-Children	1 sec	1 sec overlap	3 sec	3 sec overlap	5 sec	5 sec Overlap
Test group						
TD	0.92	0.92	0.90	0.91	0.89	0.89
CP Stan	0.90	0.91	0.89	0.89	0.87	0.88
CP Free	0.66	0.66	0.64	0.64	0.61	0.61
NTNU-HAR ChildrenCP	1 sec	1 sec overlap	3 sec	3 sec overlap	5 sec	5 sec overlap
TD	0.92	0.92	0.90	0.91	0.89	0.89
CP Stan	0.91	0.91	0.89	0.90	0.88	0.88
CP Free	0.72	0.71	0.68	0.67	0.64	0.64

The pre-processing and feature extraction

Click here to access/download;Figure;Figure1.tif

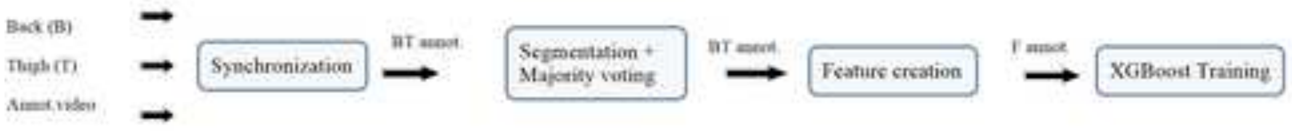

Supplement: Attachment — Submitted filename: PONE-D-23-37915_with-comments.pdf [file pone.0308853.s005.pdf]
