# Supplementary material for: Validation of two novel human activity recognition models for typically developing children and children with Cerebral Palsy
Source: PLoS One. 2024 Sep 23;19(9):e0308853. doi: 10.1371/journal.pone.0308853 (PMC11419372; doi:10.1371/journal.pone.0308853)
Supplement: Attachment — Submitted filename: Response to editor and reviewers.docx [file pone.0308853.s006.docx]

**Response to editor and reviewers.**
Our response are provided under the comments in blue, page and line numbers refer to the manuscript file with track changes.

**Journal Requirements:**

When submitting your revision, we need you to address these additional requirements.
1. Please ensure that your manuscript meets PLOS ONE's style requirements, including those for file naming. The PLOS ONE style templates can be found at 
https://journals.plos.org/plosone/s/file?id=wjVg/PLOSOne_formatting_sample_main_body.pdf and 
<https://journals.plos.org/plosone/s/file?id=ba62/PLOSOne_formatting_sample_title_authors_affiliations.pdf>
Response: Thank you for this, we have checked the guidelines again and made minor changes.
We have changed the file names of our figures after the standard. We have also changed our referral to these in the manuscript according to the guidelines. We have also changed the frontpage according to the guidelines, we have removed the authors contribution, page 2, as it seems to only be required in the submission form.

2. Please provide additional details regarding participant consent. In the ethics statement in the Methods and online submission information, please ensure that you have specified (1) whether consent was informed and (2) what type you obtained (for instance, written or verbal, and if verbal, how it was documented and witnessed). If your study included minors, state whether you obtained consent from parents or guardians. If the need for consent was waived by the ethics committee, please include this information.
If you are reporting a retrospective study of medical records or archived samples, please ensure that you have discussed whether all data were fully anonymized before you accessed them and/or whether the IRB or ethics committee waived the requirement for informed consent. If patients provided informed written consent to have data from their medical records used in research, please include this information.
Response: Thank you for pointing this out. We have updated our manuscript and the ethics statement in the submission form. Page 7, line 137-141: “All participants were given written age-appropriate information about the study. This was distributed at school and taken home together with written information and consent forms to their parents/guardians. If the parents gave their written consent, verbal information was given to the children before the study started”.

3. Please note that PLOS ONE has specific guidelines on code sharing for submissions in which author-generated code underpins the findings in the manuscript. In these cases, all author-generated code must be made available without restrictions upon publication of the work. Please review our guidelines at https://journals.plos.org/plosone/s/materials-and-software-sharing#loc-sharing-code and ensure that your code is shared in a way that follows best practice and facilitates reproducibility and reuse.
Response: Our code is a further development of previous code from our research group at NTNU. This code is already uploaded to GITHUB (<https://github.com/ntnu-ai-lab/harth-ml-experiments>). Our code includes minor changes to this code to fit the scope of our study. Up on publication our code will be provided as well.

4. We note that the grant information you provided in the ‘Funding Information’ and ‘Financial Disclosure’ sections do not match. When you resubmit, please ensure that you provide the correct grant numbers for the awards you received for your study in the ‘Funding Information’ section.
Response: We have corrected our funding information in the submission form. We do not have a specific grant number for this work. We have also updated our cover letter to include information of financial disclosure.

5. When completing the data availability statement of the submission form, you indicated that you will make your data available on acceptance. We strongly recommend all authors decide on a data sharing plan before acceptance, as the process can be lengthy and hold up publication timelines. Please note that, though access restrictions are acceptable now, your entire data will need to be made freely accessible if your manuscript is accepted for publication. This policy applies to all data except where public deposition would breach compliance with the protocol approved by your research ethics board. If you are unable to adhere to our open data policy, please kindly revise your statement to explain your reasoning and we will seek the editor's input on an exemption. Please be assured that, once you have provided your new statement, the assessment of your exemption will not hold up the peer review process.
Response: We have created a platform using Dataverse (https://dataverse.no/) where all our information regarding the data set will be published. Currently the cite is registered without the raw datafiles. However, all other metadata is provided, and the files will be uploaded on acceptance. Here is the DOI number (https://doi.org/10.18710/EPCXCC) that we refer to in our data availability statement. The DOI will be activated up on acceptance.

6. We notice that your supplementary figures are uploaded with the file type 'Figure'. Please amend the file type to 'Supporting Information'. Please ensure that each Supporting Information file has a legend listed in the manuscript after the references list.
Response: We are sorry for the mislabeling. We have changed the file type and file name according to guidelines.

Additional Editor Comments:

==============================
The reviewers have praised the work for significant contributions on human activity recognition in typically developing children and those with Cerebral Palsy but suggests improvement in addressing methodological concerns. Specific suggestions include clarifying data acquisition methods, questioning biases in training models (by both reviewer 1 and 3), and addressing discrepancies in group sizes. The reviewers 2 and 3 have common concerns about tangential literature and references used. The reviewer 1 have marked suggestions over the manuscript's pdf (see attachment), helping authors to address minor technical and langauge concerns. Finally, per the reviewer 3 suggestions, the authors are urged to discuss the implications of group sizes and consider ways to enlarge the free play dataset.
==============================
Response: We are grateful for the thoughtful comments and suggestions from the reviewers and the editorial office, which allowed us to improve our manuscript significantly. We have addressed the comments about tangential literature by providing additional references in our manuscript. We detail below how we responded to each concern from the reviewers point-by-point.

[Note: HTML markup is below. Please do not edit.]

Reviewers' comments:

Reviewer's Responses to Questions

**Comments to the Author**

1. Is the manuscript technically sound, and do the data support the conclusions?

The manuscript must describe a technically sound piece of scientific research with data that supports the conclusions. Experiments must have been conducted rigorously, with appropriate controls, replication, and sample sizes. The conclusions must be drawn appropriately based on the data presented.

Reviewer #1: Yes

Reviewer #2: Yes

Reviewer #3: Yes

2. Has the statistical analysis been performed appropriately and rigorously?

Reviewer #1: Yes

Reviewer #2: Yes

Reviewer #3: Yes

3. Have the authors made all data underlying the findings in their manuscript fully available?

The [PLOS Data policy](http://www.plosone.org/static/policies.action#sharing) requires authors to make all data underlying the findings described in their manuscript fully available without restriction, with rare exception (please refer to the Data Availability Statement in the manuscript PDF file). The data should be provided as part of the manuscript or its supporting information, or deposited to a public repository. For example, in addition to summary statistics, the data points behind means, medians and variance measures should be available. If there are restrictions on publicly sharing data—e.g. participant privacy or use of data from a third party—those must be specified.

Reviewer #1: No

Reviewer #2: Yes

Reviewer #3: Yes

4. Is the manuscript presented in an intelligible fashion and written in standard English?

PLOS ONE does not copyedit accepted manuscripts, so the language in submitted articles must be clear, correct, and unambiguous. Any typographical or grammatical errors should be corrected at revision, so please note any specific errors here.

Reviewer #1: Yes

Reviewer #2: Yes

Reviewer #3: Yes

5. Review Comments to the Author

Please use the space provided to explain your Responses to the questions above. You may also include additional comments for the author, including concerns about dual publication, research ethics, or publication ethics. (Please upload your review as an attachment if it exceeds 20,000 characters)

**Reviewer #1:**
Typically developed children. Overall, on the technical side, the presented manuscript is technically sound, and all the result data support the authors' claims in the result and conclusion sections. The authors' choice of F-1, understanding of F-1, and usage are respectable. Under the microscope, however, there are minor editorial/technical errors—for example, the authors' inconsistent citation style or difficulty in explaining their 12 models concept. Furthermore, the authors haven't made any data available for public consumption. Please refer to the reviewer attachment for a complete list of questions/comments in the form of notes embedded in the manuscript PDF. Overall, the reviewer concluded that this manuscript is legible and technically sound, and all the analyses have been done appropriately.

Response:
Thank you for your comments and feedback. We have made changes accordingly.

- Regarding the F1 Score, see point 9 in the specific comment section bellow.
- Our citations and references are corrected according to Vancouver reference style.
- We have made several changes to the document regarding the 12 sub-model explanation, which will make it easier for the reader to follow our argumentation. See new supporting figure 4 (S4 Fig) and revised manuscript on page 10, line 222-224. “The two main models comprise a total of 12 sub-models, 6 for each main model, covering 1-, 3-, and 5-seconds windows with and without overlapping windows, see supporting information (S4 Fig) for overview of the 12 sub-model creation”.
- Regarding the data availability we have created a cite on Dataverse where the data will be published upon exception/publication (https://doi.org/10.18710/EPCXCC). Our code can be found with similar codes from our research team at GITHUB: https://github.com/ntnu-ai-lab/harth-ml-experiments.

Comments from the manuscript file:

1. Line 79. Missing definition.
   Response: Thank you for this comment. Definition of TD is typically developing, we have made a typo on page 4 line 76. We have now corrected the typo and the definition is there.
2. Line 80. This notion is in conflict with previous statement.

Response: We agree that the sentences needed adjustments. We have rewritten the paragraph.

Page 4/5, line 81-86.

3. Line 89. Citations?

Response: We have rewritten the sentence and added references for the updated prevalence. Page 5, line 96-98. “CP is one of the most frequent disability among children (25) *with the global prevalence of approximately 1.6 /1000 live births in high income countries (26).”*

4. Line 99. Wrong citation style.

Response: We have corrected the format.

5. Line 172-174. Was the labelling done with one person or more? If more, an addition of Cohen's kappa agreement coefficient would be beneficial in justifying the dataset’s quality by looking at the reliability between the rater(labeller).

Response: More than one person labelled the data. In Ustad et al., (2023) and Bach et al., (2022) they have reported interrater reliability, with the coefficient of >0.95. Using the same method and activity definitions and overlapping raters as our study. We have added this information in our manuscript on page 9, line 189-191.
Ustad A, Logacjov A, Trollebø SØ, Thingstad P, Vereijken B, Bach K, et al. Validation of an Activity Type Recognition Model Classifying Daily Physical Behavior in Older Adults: The HAR70+ Model. Sensors. 2023;23(5):2368.

Bach K, Kongsvold A, Bårdstu H, Bardal EM, Kjærnli HS, Herland S, et al. A Machine Learning Classifier for Detection of Physical Activity Types and Postures During Free-Living. Journal for the Measurement of Physical Behaviour. 2022;1(aop):1-8.

6. Line 183. Why 50% overlap?

Response: When employing HAR models with fixed windows there is a potential loss of some activities at the endpoint of the window. The choice of a 50% overlap was deliberate to ensure that no single endpoint in the data was overlooked as the new window would be in the middle of the previous window. The 50% overlap is commonly used in HAR, when employing these fixed windows. We considered increasing the overlap, however, as it would substantially increase the processing time it will make the model unsuitable for clinical purposes. Under we have provided two references that also use 50% overlap in their HAR models. We have added a sentence on this in our manuscript on page 9, line 199-200. “The overlap was included to investigate potential loss of activities at the endpoint of the window”.


Lara OD, Pérez AJ, Labrador MA, Posada JD. Centinela: A human activity recognition system based on acceleration and vital sign data. Pervasive and mobile computing. 2012 Oct 1;8(5):717-29.

Ferrari A, Micucci D, Mobilio M, Napoletano P. On the personalization of classification models for human activity recognition. IEEE Access. 2020 Feb 12;8:32066-79.

7. Line 191. Pleas don’t forget to cite the original XGBoost paper: <https://arxiv.org/abs/1603.02754>
   Response: Thank you for providing this reference for us. We have cited at page 10, line 208.
8. Line 201-202. A table or a more descriptive definition, whether in the text or the supplement section, would help greatly digest this sentence.

Response: We agree and have provided a figure in the supplementary information (S4 Fig) that explain how we get to 12 sub models and how these are linked. We have revised our manuscript page 10, line 217-224.

9. Line 204 and 205. Why use k-fold here and LOSOCV in the other section? A K-fold CV might have higher accuracy but is more likely to be artificial due to intra-subject contamination, unlike LOSOCV. Additionally, leave-n-subject-out cross-validations might be more beneficial as they provide a cross-subject generalisability (subject-independent performance) overview while maintaining a manageable number of cross-validation-round.
   Response: Regarding the use of K-fold or LOSOCV we have only used the method of LOSOCV. We have used the LOSOCV method as a k-fold on 6 subjects, or as you suggested leave-n-subjects-out-cross-validation. We have revised the manuscript, and we are sorry for the confusion. Modifications on page 10, line 224-225. “For each model we initially performed a leave-six-subjects-out-cross-validation with hyperparameter optimization in the form of a grid search”.
10. Line 239. Are they class weighted or unweighted?

Response: We have calculated macro/ non-weighted F1 Score using the following formula F1 Score= 2x precision x recall / precision + recall. As we only report class-wise F1 Scores we have concluded that there is no need for calculating the weighted F1 Score in our results. Updated manuscript on page 12, line 265.

**Reviewer #2:** 
Authors have presented an ML-based Human Activity Recognition (HAR) model for typically developing (TD) children and children with Cerebral Palsy (CP).
The study discusses the advantages of HAR models for the same and challenges in the setup and hyperparameters such as training data, optimal accelerometer placements,
and window size considerations. It presents the usage of the XGBoost classifier (12 sub-models) with 1, 3, and 5 sec time windows. The ML-based approach [1, 2, 3] is a very common HAR.
Response: Thank you for your comments on our manuscript and suggestions for further work and improving the usability of human activity recognition models. Additionally, we would thank you for the thorough work of including references to your comments. We have provided our response to your comments bellow.

Following are my comments to further improve this article:

1. The applicability of the study may be broadened by including the usage of HAR in different health and rehabilitation fields such as exercise/YOGA pose classification [4], rehabilitation of individuals after sports injury, and improving the posture for the different exercises and training.
Response: Yes, we agree that the study might be broaden by including additional activities. As we aimed to investigate children’s activity, we tried to include childlike behavior and prioritize this in our training. We initially want to identify the basic movements and postures. Inclusion of e.g. yoga poses would be an interesting option for future endeavors in model training of complex activities.

2. The study can also include a) a comparison of deep learning-based models [3, 4, 6] along with ML-based models for the HAR model for TD and CP children and b) a study of multimodal features [6] in HAR. Authors may refer to the studies [1, 5, 6] for choosing appropriate ML and DL methods for the task.
Response:
a) It is a good suggestion to compare different types/ methods of computer sciences. We have discussed running our experiments using deep learning methods, however we are uncertain if this will improve the model performance, as our ML models are very good. We have concluded that running additional experiments and evaluation different methods is beyond the scope of our study. We have also gotten feedback that our study has a lot of models and results, therefore we have concluded that adding additional methods would be excessive and not contribute significantly to our research question. Nevertheless, we thank you for your suggestion and note it as a future research endeavor to perform deep learning experiments.
b) Ideally, we would like to study multimodal features. However, as we aim for research regarding children, one of the main ethical considerations is that we cannot put excessive amounts of equipment on children. Accelerometers are the preferred method for activity recognition and placing one or two accelerometers on a child will give minimal burden for the research participants or patients. Therefore, for our objective and feasibility in habilitation multimodal features are beyond the scope of our study.

3. The author should consider the experimentation of multiple related datasets such as WISDM Activity Prediction, UCI HAR, DSADS, etc. Please refer to Table 1 from [6] for a list of publicly available datasets of HAR.
Response: Thank you for this list of data sets and the reference number 6, which include additional publicly available data sets. Unfortunately, all these data sets are on adults or older individuals with movement restrictions. Therefore, we cannot use these to train a model for children and enhance our model. However, this emphasizes our objective to create this data set and model for TD and CP children.

4. The possible direction to measure the automatic improvement in CP children after the rehab/therapy would further boost the applicability of research in a real-world application.
Response: This is a very important perspective. To use the HAR models for this purpose is one of our main objectives for creating and validating our model. Further in our project we are using our HAR model to evaluate the activities children with CP engage in. In our next study we aim to describe the activity patterns and distribution of activity in children with CP. And hopefully in the future implement our HAR-model in the clinical setting and as you suggest; evaluate treatment effects in habilitation of children with CP. We have made some changes to our manuscript on page 26, line 509-511 to emphasize this possibility for the future.

5. In line 73 of the Introduction section, TD is mistyped as TP.
Response: Thank you for noticing this, we have corrected the typo.

6. In line 243 of section 2.6, it is written as F-measure is the harmonic mean between precision and sensitivity, whereas it is the harmonic mean between precision and recall [7].
Response: We have discussed this and changed the manuscript to include both sensitivity and recall, as computer scientists often use recall and clinicians often use sensitivity to describe the true positive rate. See page 12, line 261 for the adjustment.

References from reviewer:
1. Gupta, N., Gupta, S.K., Pathak, R.K. et al. Human activity recognition in artificial intelligence framework: a narrative review. Artif Intell Rev 55, 4755–4808 (2022). https://doi.org/10.1007/s10462-021-10116-x
2. Csizmadia, G., Liszkai-Peres, K., Ferdinandy, B. et al. Human activity recognition of children with wearable devices using LightGBM machine learning. Sci Rep 12, 5472 (2022). <https://doi.org/10.1038/s41598-022-09521-1>
3. D. Sakkos, K. D. Mccay, C. Marcroft, N. D. Embleton, S. Chattopadhyay and E. S. L. Ho, "Identification of Abnormal Movements in Infants: A Deep Neural Network for Body Part-Based Prediction of Cerebral Palsy," in IEEE Access, vol. 9, pp. 94281-94292, 2021, doi: 10.1109/ACCESS.2021.3093469.
4. Vallabhaneni, N., Prabhavathy, P. Segmentation quality assessment network-based object detection and optimized CNN with transfer learning for yoga pose classification for health care. Soft Comput (2023). https://doi.org/10.1007/s00500-023-08863-w
5. Wan, S., Qi, L., Xu, X. et al. Deep Learning Models for Real-time Human Activity Recognition with Smartphones. Mobile Netw Appl 25, 743–755 (2020). https://doi.org/10.1007/s11036-019-01445-x
6. Kaixuan Chen, Dalin Zhang, Lina Yao, Bin Guo, Zhiwen Yu, and Yunhao Liu. 2021. Deep Learning for Sensor-based Human Activity Recognition: Overview, Challenges, and Opportunities. ACM Comput. Surv. 54, 4, Article 77 (May 2022), 40 pages. https://doi.org/10.1145/3447744
7. Schütze, H., Manning, C. D., & Raghavan, P. (2008). Introduction to information retrieval (Vol. 39, pp. 234-265). Cambridge: Cambridge University Press.

**Reviewer #3:** 
The article “Validation of two novel human activity recognition models for typically developing children and children with Cerebral Palsy” is well written and pleasurable to read. It presents a classifying method of physical activities in CP children and above all the influence of different parameters of this method: learning dataset composed of only TD children or a mix of TD and CP children, the length of the observation window, the choice to use or not an overlap to define this window. Results are clear and complete which helps to understand the effect of each parameter. Collecting and processing the dataset has been a very consequent job that might be very useful for any research team working on physical activity recognition in CP children in real life which is a crucial question. I would like to sincerely thank the authors for that huge and useful work.

Nevertheless, some methodological choices and the ensuing elements of discussion should be addressed to strengthen the impact of this study.

Response: Thank you for your positive comments and remarks on our manuscript. Our response to your comments and changes in our manuscript are listed point-by-point below.

Specific comments

L44: As soon as the abstract you should indicate how data are acquired (video, IMU, … ?)
Response: We have updated our abstract and established how we collected data and annotated on page 3 line 48-49. “Our data was collected using accelerometers and ground truth was established with video annotations.”

L85: It is a bit surprising that in most of previous studies, the authors did not think that training the model with one population (adults) would be a bias when using the model with another one (children). This bias is presented as on major argument for your study. However, I am pretty sure this potential bias is largely exaggerated as in most studies aiming at recognizing human activities the training population has the same characteristics as the target one.
Response: We agree with your comment that most studies train the model with data sets from their target populations. However, from our literary search we see that most models are developed on heathy adults, probably because human behavior studies often target this population. Additionally, most models are developed on adult activities, like cleaning the house, making food and not play activities. Moreover, the movement patterns for children with disability like CP, is different from that of TD children. Consequently, this creates a call for our model, that is built on and for TD children and children with CP. We have clarified our perspective in the manuscript on page 4-5, line 81-96.

L99: And even, the three articles you present come from the same research team in Queensland, Australia. I am very surprised that only two research teams in the world are interested in PA recognition in CP. Are you only focusing on the articles in which the methodology used for this recognition is very close to yours so that comparison is easier? If so that needs to be modified. You should be able to compare your results even when the classifier’s principle is different from yours.
Response:
- We have conducted a systematic literature search. We have provided you the search history and Prisma chart at the end of this file. In our search we included different types of HAR models, including both ML models and deep learning. Our search details will show that we have opted to include all previous research.
- Our aim is not to compare methodology; therefore, we have only included the studies that use the same methodology, meaning ML models. Within the ML methodology we have included all types of classifiers, window settings, feature extractions and so on.
- From our search we only found four studies that included children with CP, one of them is from a different research group than the one in Queensland and include children with disabilities. We have now included this article in the manuscript page 6, line 110 and 113.


L115: It is not clear what are the ‘two’ models. There is no prior justification for the need of two different models and what will be the advantage of one vs. the other, or in which cases one should be used. Just after, I understand that you mean two models because learning data are not the same, e.g. one with only TD and the other one mixing TD and CP. The question is to know if the methodology is identical in both cases. If it is, I would rather say that there is actually only one model with which you are testing the influence of identifying the model’s parameters from two learning datasets. Indeed, afterwards you are talking about 12 models when combining this learning dataset parameter to two other ones, i.e. length of window (1, 3 or 5 s) and the overlap.
**Response:** We understand this confusion and have clarified it with a new figure in the supplementary information, S4 Fig. We have used one methodology, building two different models. The training data is what makes them different. We are sorry that this was somewhat unclear, and have improved text page 10, line 217-224. We have also adjusted our justification for two models in the introduction and on page 6, line 114/115.


L123: The two groups have very different size (ratio 1:4). It means that in your second dataset, mixing TD and CP, TD activities are actually the large majority. On L201, you don not indicate if the second dataset includes all data from TD children. I thus assume that it is the case. It actually means that your second ‘model’ center of gravity is actually drifted towards TD movements.
Response:
- We have made adjustments to clarify that the ChildrenCP model also included all TD training data. “.. and the HAR-ChildrenCP was trained with all TD training data and additional data from children with CP” page 10, line 221. This clarification is also reflected in our new supplementary figure S4 Fig.
-We acknowledge that our CP model consist of more TD training data than CP. However, following the principle that more training data and variation in movement patterns generally benefits model training we opted to include all TD data in the CP model. Our results show that including CP data increase the model performance. We have adjusted the manuscript on page 25, line 485-491.

L155: What is the sensitivity of your method to accelerometer misplacement? The final goal is to monitor activities in real life situation in which CP children or their parents will place the devices. They are not clinicians trained to the use of such device. One usual error in that case is axes misalignment. It is important to test this robustness to that kind of bias. In your experiment, these biases were absent as all the devices were placed by the team (maybe even the same operator). It implies that the data used in the LOSOCV evaluation is homogeneous regarding these biases. As being outside the learning database, the child left-out represents a subject in real life. But sensors’ placement on a child in real life will be less precise.
Response: Thank you, this is a relevant question. Our research group have tested the consequence of sensor misplacement on model performance in previous studies using the same methodology (unpublished). As long as the sensors are placed in the correct direction towards gravity minor deviations in placements does not affect the accuracy of the model.

L197: Fig. 1 is hardly readable.
Response: Thank you for noticing this. We have created a new Fig 1.

Discussion: you should discuss a bit more about the groups’ sizes. I agree with you that your whole dataset with 79 children is quite large compared to other studies. But 80% of them are TD. And moreover, as some activities (e.g. jumping) is more difficult for CP children, when focusing more finely on the dataset activity per activity, this ratio is sometimes even more in favour of TD children.
Response: There is also variation in movement patterns within the TD population. Thus, we do not think that the ratio in favor towards TD children will substantially influence the model performance. However, adding more CP data would probably improve the model performance further, due to even more variation in the training data’s movement patterns. Page 25, line 485-491.

L462: I agree with you that it is always a bit frustrating not to have more free play datasets. Free play, even in clinical environment, is one important way to be closer to real life. You honestly recognize that your free play dataset should be enlarged but not indicating how this could be done. If you have an idea, could you explain it briefly?
Response: On page 25, line 504-508 we have discussed how to further enhance the applicability of the models with including data from real world activities. We have modified the paragraph to indicate that this is to enlarge the free play data set.

6. PLOS authors have the option to publish the peer review history of their article ([what does this mean?](https://journals.plos.org/plosone/s/editorial-and-peer-review-process#loc-peer-review-history)). If published, this will include your full peer review and any attached files.


If you choose “no”, your identity will remain anonymous but your review may still be made public.


**Do you want your identity to be public for this peer review?** For information about this choice, including consent withdrawal, please see our [Privacy Policy](https://www.plos.org/privacy-policy).

Reviewer #1: No

Reviewer #2: **Yes:** Dr. Durgesh Kumar

Reviewer #3: No

**Search details for a systematic literature search that provided us with the three(four) articles that includes children with CP and HAR-models. Including the Prisma-flow chart.**

| **Inclusion criteria** | **Exclusion criteria** |
| --- | --- |
| Use machine learning on raw acceleration signal  Children with Cerebral palsy  English or Scandinavian language  Primary paper with original data | Use cut-point or counts-per-minute before activity recognition  Master thesis  Reviews  Off-topic for literature search |
