# Supplementary material for: Validation of two novel human activity recognition models for typically developing children and children with Cerebral Palsy
Source: PLoS One. 2024 Sep 23;19(9):e0308853. doi: 10.1371/journal.pone.0308853 (PMC11419372; doi:10.1371/journal.pone.0308853)
Supplement: Attachment — Submitted filename: Response to Reviewers.docx [file pone.0308853.s007.docx]

**Response to editor and reviewers.**
Our response is provided under the comments in blue, page and line numbers refer to the manuscript file with track changes.

PONE-D-23-37915R1

Validation of two novel human activity recognition models for typically developing children and children with Cerebral Palsy.

PLOS ONE

Dear Dr. Tørring,

Thank you for submitting your manuscript to PLOS ONE. After careful consideration, we feel that it has merit but does not fully meet PLOS ONE’s publication criteria as it currently stands. Therefore, we invite you to submit a revised version of the manuscript that addresses the points raised during the review process.

Please submit your revised manuscript by May 16, 2024, 11:59PM. If you will need more time than this to complete your revisions, please reply to this message or contact the journal office at [plosone@plos.org](mailto:plosone@plos.org). When you're ready to submit your revision, log on to https://www.editorialmanager.com/pone/ and select the 'Submissions Needing Revision' folder to locate your manuscript file.

Please include the following items when submitting your revised manuscript:

- A rebuttal letter that responds to each point raised by the academic editor and reviewer(s). You should upload this letter as a separate file labeled 'Response to Reviewers'.
- A marked-up copy of your manuscript that highlights changes made to the original version. You should upload this as a separate file labeled 'Revised Manuscript with Track Changes'.
- An unmarked version of your revised paper without tracked changes. You should upload this as a separate file labeled 'Manuscript'.

If you would like to make changes to your financial disclosure, please include your updated statement in your cover letter. Guidelines for resubmitting your figure files are available below the reviewer comments at the end of this letter.

If applicable, we recommend that you deposit your laboratory protocols in protocols.io to enhance the reproducibility of your results. Protocols.io assigns your protocol its own identifier (DOI) so that it can be cited independently in the future. For instructions see: <https://journals.plos.org/plosone/s/submission-guidelines#loc-laboratory-protocols>. Additionally, PLOS ONE offers an option for publishing peer-reviewed Lab Protocol articles, which describe protocols hosted on protocols.io. Read more information on sharing protocols at <https://plos.org/protocols?utm_medium=editorial-email&utm_source=authorletters&utm_campaign=protocols>.

We look forward to receiving your revised manuscript.

Kind regards,

Jyotindra Narayan
Academic Editor
PLOS ONE

**Additional Editor Comments:**

==============================
Reviewer #1 recommends accepting the manuscript, acknowledging its contribution to Human Activity Recognition (HAR) in typically developing children and children with cerebral palsy. While noting improvements in presentation and writing, Reviewer #2 raises concerns about the lack of novelty in methodology and research objectives. Suggestions for enhancement include incorporating a deep learning-based model for comparison with XGBoost, utilizing multimodal features combining video image and sensor data, sharing data with the research community, and addressing how the proposed models differ from existing methods. Reviewer #3 appreciates the authors' responses to concerns but suggests including unpublished results regarding sensor misplacement's impact on model performance as a potential limitation.
==============================

**Response to editor:**
Dear Jyotindra Narayan

We would like to express our gratitude for the thorough review process and the insightful comments provided by the reviewers and the editorial office on our manuscript. We appreciate the time and effort invested by all involved, which has undoubtedly contributed to the improvement of our work. We have carefully considered each comment and suggestion raised by the reviewers and have made necessary revisions to our manuscript accordingly.
Regarding the suggestions put forth by Reviewer #2, we respectfully acknowledge the reviewer's perspective and the importance of exploring various avenues for advancement in Human Activity Recognition (HAR). However, we must clarify that our primary objective is to develop and validate a HAR model tailored to typically developing children and children with cerebral palsy, with deviating movement pattern, by using existing HAR methodology. Our objective was not to compare two methodologies for activity recognition but validate two models.
 Reviewer 2 has raised a valid point regarding the novelty of our study's method and objectives. We acknowledge that our study does not introduce significant novelty in terms of methodology, as there already exist Human Activity Recognition (HAR) models employing various machine learning techniques. However, it is worth noting that few of these models are built on data from children, and only three focus on children with cerebral palsy (CP).
 From our perspective novelty in our study is evident in several aspects. Firstly, the uniqueness of our data set stands out, as it includes a substantial number of typically developing (TD) children alongside children with CP. This diverse data set provides a rich foundation for our analysis. Secondly, the inclusion of free play activities, including childlike activities and ball games, adds a novel dimension. These activities reflect real-world scenarios and add depth to our understanding of activity recognition in children. Thirdly, our comparison between TD and CP models is unprecedented, particularly testing a model designed for children with deviating movement patterns on typically developing children. Lastly, our exploration of window sizes and overlap also contributes valuable insights to the field, by offering insights into optimal parameter settings for activity recognition systems.
 In addition to our points raised in the discussion regarding future perspectives and implications, we acknowledge the need for further improvements to HAR methodology. In response to Reviewer 2's suggestion, we have included a new paragraph under this heading about deep learning as a potential avenue for future optimization of HAR in general. We fully recognize the value of further exploration of different methodologies as deep learning, but we believe they warrant dedicated studies of their own. Therefore, we kindly request your understanding in accepting our manuscript without additional experiments that incorporating multimodal features and deep learning-based models.
Once again, we extend our sincere appreciation to the reviewers and the editorial office for their valuable feedback and consideration of our work. We remain committed to contributing to the advancement of knowledge in the field of Human Activity Recognition and look forward to the opportunity to share our findings with the research community.

Thank you for your attention to this matter.

Yours sincerely,

Marte Fossflaten Tørring
NTNU

- Review Comments to the Author

  Please use the space provided to explain your answers to the questions above. You may also include additional comments for the author, including concerns about dual publication, research ethics, or publication ethics. (Please upload your review as an attachment if it exceeds 20,000 characters)

**Reviewer #1**:
(No Response)

**Reviewer #2:** 
The manuscript solves a real-world problem of Human Activity Recognition (HAR) in normal children and children with celebration palsy. The authors have improved in presentation and writing from the first revised version. However, the manuscript lacks novelty in terms of the proposed methodology and research Objectives. The significant research contributions of the study are as follows:
1. The novel datasets for Human Activity Recognition for Typically Developing Children (TD) and Children with Cerebral Activity.
2. Experiment and Analysis of HAR on the above datasets using XGBoost classifier.
3. Study of the impact of windows size (1 sec, 3 sec, 5 sec) and with overlapping.

In my first review, I have provided possible direction to extend the novelty and technical contribution of the paper by including:
1. Inclusion of a Deep learning-based model (such LSTM) for comparison with XGBoost: The author refused to experiment with the model, citing their assumption that it would not improve their results further without even experimenting with it. LSTM and BiLSTM are known to improve the results in sequential data.

**Response:**
Thank you for the time and effort you spent on our manuscript. We appreciate your thoughtful consideration of our work and your suggestions for improvements. We acknowledge that our study does not introduce significant novelty in terms of technical methodology, as there already exist Human Activity Recognition (HAR) models employing various machine learning techniques. However, it is worth noting that few of these models are built on data from children, and even fewer focus on children with cerebral palsy (CP).
 From our perspective novelty in our study is evident in several aspects. Firstly, the uniqueness of our data set stands out, as it includes a substantial number of typically developing (TD) children alongside children with CP. This diverse data set provides a rich foundation. Secondly, the inclusion of free play activities, including childlike activities and ball games, adds a novel dimension. These activities reflect real-world scenarios and add depth to our understanding of activity recognition in children. Thirdly, our comparison between a TD and CP model is unprecedented, particularly testing a model designed for children with deviating movement patterns on typically developing children. Lastly, our exploration of window sizes and overlap also contributes to valuable insights to the field, by offering insights into optimal parameter settings for activity recognition systems. Our focus stems from our commitment to addressing clinical needs and facilitating activity identification in children with CP. We have clarified this further in the manuscript.
 We apologize for giving the impression that we didn't think the suggested models would have any potential improving activity detection. We acknowledge that including additional experiments, as you suggest, could potentially yield some improved results and novelty in terms of methodology. We have included a new paragraph under the heading future perspectives and implications about deep learning as a potential avenue for future optimization of HAR in general (page 25, line 485-492). We fully recognize the value of further exploration of different methodologies as deep learning, but we believe they warrant dedicated studies of their own. Therefore, in our paper, we adhere to the original scope, objectives, and methods of our study. We have clarified our objective on page 5, line 95-102. We hope this clarification resonates with your understanding of our research goals and constraints.

Further, the authors claimed there was a lack of space to fit their results, as they had too many large result tables. The results table could appropriately be compressed by representing it with suitable diagrams.
**Response:**
We apologize for the words chosen in our previous answer and we thank you for the suggested solution. However, our decision not to expand the experiments beyond the scope of our current study was based on several factors, including the choice to maintain focus on our research question within the clinical context. Introducing further experiments is not solely restricted to space limitations but also risks complicating the manuscript for readers, particularly those outside the technological community.
Moreover, we would like to refer to our answer to your first remark; that expanding the scope of experiments would necessitate the establishment of new objectives. Given our commitment to ensuring accessibility and understanding, especially among clinicians and other stakeholders in the clinical community, we must prioritize clarity and conciseness in our presentation.

2. Inclusion of multimodal features: I mean to use both the video image and sensor data for the HAR. As the confusion matrix (Figure 5) NNTU HAR ChildrenCP-5 sec shows, some of the Walking and running data is wrongly predicted as Standing. In such a scenario, the image taken from the camera could assist the sensor data in proper activity recognition.
**Response:**
It seems we may have misunderstood each other, and we apologize for any confusion.
In our study, we aim to further develop and validate the use of small wearable sensor technology to monitor activity in everyday life of children with cerebral palsy. In our study, the video recordings are used as a “gold standard” which the model is tested against. If we understand correctly, you're proposing the utilization of video recordings from the children to enhance the model's performance. While integrating multimodal features as you suggest could undoubtedly optimize the activity recognition model, we must consider the practical constraints within our clinical context.
Incorporating 24/7 video recordings from children's everyday lives would present several challenges, including privacy concerns, ethical considerations, and increased burden on the patients and is therefore not feasible.

3. The authors have highlighted the lack of publicly available data for HAR in children and children with CP. Unless researchers share their data for academic and research purposes, this problem will remain as it is. Therefore, it is requested that the authors share their data with the research community after the journal accepts it. Please share the data sharing policy and some data samples with the Editorial Team before the publication of the papers.
**Response:**
We totally agree with the importance of data sharing in advancing research efforts. In response to this concern, we have developed a comprehensive data sharing plan, which we previously shared with the editorial staff and included in our response to reviewer 1 during the previous review round. Here is a recap of our data sharing plan:

Our data will be made publicly available upon acceptance/publication through a dedicated repository on Dataverse, accessible via the following link: https://doi.org/10.18710/EPCXCC. Additionally, we have ensured that our codebase is readily accessible for fellow researchers and interested parties. You can find our code, along with similar codes from our research team, on our GitHub repository: https://github.com/ntnu-ai-lab/harth-ml-experiments.
 We are committed to fostering collaboration and contributing to the collective advancement of knowledge in this field. We appreciate your attention to this matter and your dedication to promoting open science principles. We will share data with the editorial office upon request from the editor.

4. How much do the Model NNTU-HAR-Children and NNTU-HAR-ChildrenCP differ from the NNTU-HAR method proposed by Roar M. Fenne?
**Response:**
Our models, NTNU-HAR-Children and NTNU-HAR-ChildrenCP, are developed using training data from children and adolescents, and are tailored to recognized activity patterns often seen in children. The master’s thesis by Roar M. Fenne tested how well a model trained with data from adults performed in recognizing activities in children. For more information about the adult model used in the study by Fenne, I recommend exploring the recent publication titled "A Machine Learning Classifier for Detection of Physical Activity Types and Postures During Free-Living" by Bach et al. 2022.

.
Other Minor comments:
1. In section 2.4.2 (Video Annotation), it is not clear how many annotators were there in total, and each video frame annotated by how many annotators, and what was the score inter-annotator agreement?
**Response:**
Thank you for noting this unclarity in section 2.4.2 regarding the video annotation process. We have considered your suggestion and updated our manuscript accordingly. On page 8, lines 161-165 now provide more clarity on the annotation process:

“*After clarifying the activity definitions and discussions based on video examples four raters annotated each video independently. Inter-rater reliability of > 0.95 on this methodology has been reported in earlier studies using the same methods, activity definitions and overlapping raters with the present study (Bach et al., 2022; Ustad et al., 2023).”*

Bach, K., Kongsvold, A., Bårdstu, H., Bardal, E. M., Kjærnli, H. S., Herland, S., Logacjov, A., & Mork, P. J. (2022). A Machine Learning Classifier for Detection of Physical Activity Types and Postures During Free-Living. *Journal for the Measurement of Physical Behaviour*, *1*(aop), 1-8.

Ustad, A., Logacjov, A., Trollebø, S. Ø., Thingstad, P., Vereijken, B., Bach, K., & Maroni, N. S. (2023). Validation of an Activity Type Recognition Model Classifying Daily Physical Behavior in Older Adults: The HAR70+ Model. *Sensors*, *23*(5), 2368.

2. The research has not concluded the optimal time window for the HAR in their conclusion section and results discussion section.

**Response:**
Thank you for pointing this out.
In our results discussion section, we acknowledge the importance of the time window size, and we conclude that it is dependent on the target activity. Specifically, we highlight that the two 1-second models outperform others across test groups and most activities, except for cycling, where 3- or 5-second windows show better performance. Page 18-19, lines 332-335 and 337-340. We would also like to refer to the section where we discuss the window settings and the preference of different time windows on page 21-22, lines 403-440.

We have revised our conclusion to provide a more precise summary of our findings. On page 25, lines 499-507 now state:

"*Based on the highest overall accuracy, the NTNU HAR-ChildrenCP model with 1-second window would be recommended for all three test groups. However, the optimal window size and overlap depend on the target activity. For activities such as cycling, 3- or 5-second windows perform better. Considering the ability to predict both momentary activities and activities of extended duration, the 3-second window without overlap would be recommended for population measurements.*"

**Reviewer #3:**
The authors provided convincing answers to my concerns and consequently modified the article. There is only one concern for which extra information would be useful. Regarding robustness to sensors' misplacement, the authors explained that "Our research group have tested the consequence of sensor misplacement on model performance in previous studies using
the same methodology (unpublished). As long as the sensors are placed in the correct direction towards gravity minor deviations in placements does not affect the accuracy of the model." Even if unpublished yet, the results of these previous studies could be presented in discussion as a potential limitation already taken into account. In the current version, it is as if the limitation does not exist.
**Response:**
Thank you for your positive feedback and for recognizing the modifications we've made to address your concerns. We acknowledge the importance of transparency regarding potential limitations, including sensor misplacement, in our study.
 In response to your suggestion, we've updated our manuscript to include this information in the discussion section. On page 24, lines 481-484 now state:

"*A potential limitation for future clinical use is sensor misplacement, as it is crucial to ensure correct alignment with gravity for accurate data collection. Therefore, it's important to educate participants on proper sensor placement, especially when positioning sensors outside the clinic*."

We believe this addition provides valuable context for readers and highlights our awareness of potential limitations.
