# Supplementary material for: Validation of two novel human activity recognition models for typically developing children and children with Cerebral Palsy
Source: PLoS One. 2024 Sep 23;19(9):e0308853. doi: 10.1371/journal.pone.0308853 (PMC11419372; doi:10.1371/journal.pone.0308853)
Supplement: Attachment — Submitted filename: Response to reviewers.docx [file pone.0308853.s008.docx]

**Response to editor and reviewer**

Our response is provided in blue under the comments, page and line number refer to the manuscript file with track changes.

Dear Dr. Tørring,

Thank you for submitting your manuscript to PLOS ONE. After careful consideration, we feel that it has merit but does not fully meet PLOS ONE’s publication criteria as it currently stands. Therefore, we invite you to submit a revised version of the manuscript that addresses the points raised during the review process.

Despite the reviewers agreed about the significant revisions made by the authors, they are still not convinced on the novelty and technical contribution of the work. The authors should present the model file and include some results for deep learning-based experiments in the manuscript to improve the technical contributions of the work adequately. 

Please submit your revised manuscript by Aug 08 2024 11:59PM. If you will need more time than this to complete your revisions, please reply to this message or contact the journal office at [plosone@plos.org](mailto:plosone@plos.org). When you're ready to submit your revision, log on to <https://www.editorialmanager.com/pone/> and select the 'Submissions Needing Revision' folder to locate your manuscript file.

Please include the following items when submitting your revised manuscript:

- A rebuttal letter that responds to each point raised by the academic editor and reviewer(s). You should upload this letter as a separate file labeled 'Response to Reviewers'.
- A marked-up copy of your manuscript that highlights changes made to the original version. You should upload this as a separate file labeled 'Revised Manuscript with Track Changes'.
- An unmarked version of your revised paper without tracked changes. You should upload this as a separate file labeled 'Manuscript'.

If you would like to make changes to your financial disclosure, please include your updated statement in your cover letter. Guidelines for resubmitting your figure files are available below the reviewer comments at the end of this letter.

If applicable, we recommend that you deposit your laboratory protocols in protocols.io to enhance the reproducibility of your results. Protocols.io assigns your protocol its own identifier (DOI) so that it can be cited independently in the future. For instructions see: <https://journals.plos.org/plosone/s/submission-guidelines#loc-laboratory-protocols>. Additionally, PLOS ONE offers an option for publishing peer-reviewed Lab Protocol articles, which describe protocols hosted on protocols.io. Read more information on sharing protocols at <https://plos.org/protocols?utm_medium=editorial-email&utm_source=authorletters&utm_campaign=protocols>.

We look forward to receiving your revised manuscript.

Kind regards,

Jyotindra Narayan
Academic Editor
PLOS ONE

**Response to editor:**
Dear editor, Jyotindra Narayan

Thank you for your understanding and for your considerate suggestion. We appreciate the time and effort you have dedicated to evaluating our manuscript and guidance in the current situation. We would like to refer to our email correspondence for further information about our position.

While we have decided not to engage in an extensive round of revisions that would alter the original scope of our study, we will proceed with making the minimal revisions as recommended.

Specifically, we will:

1. Clarify the novelty of our work, once again emphasizing the clinical relevance of our study focused on children with CP.
2. Provide the model file trained on all subjects to enhance reproducibility on GitHub.

We have also made changes to our manuscript to highlight these points.

Finally, we would once again emphasize that two of the reviewers have already accepted our manuscript. Therefore, we ask you to make an editorial decision based on all three reviewers’ remarks. Thank you once again for your guidance and support. We look forward to your final decision.

Best regards,

Marte Fossflaten Tørring

6. Review Comments to the Author

Please use the space provided to explain your answers to the questions above. You may also include additional comments for the author, including concerns about dual publication, research ethics, or publication ethics. (Please upload your review as an attachment if it exceeds 20,000 characters)

Reviewer #2: The manuscript lacks significant technical contribution except for noble datasets. However, there had been few publicly available HAR datasets ( but not with children and cerebral palsy). Several works have already been on HAR for different age groups and people with cerebral palsy. However, as per the author's claim, this is the first work on HAR in children with Cerebral Palsy.

We appreciate your recognition of the unique dataset we have presented.

As we mentioned in our previous response, we see our novelty not in technical innovation but in the utilization of a technical methods in real-world scenarios and clinical settings. We would like to clarify a few points regarding the novelty and technical contribution of our work:

1. **Not the First HAR Model for Children or Children with CP**: While it is true that there have been previous HAR models for children and individuals with Cerebral Palsy, our work differentiates itself by achieving a level of accuracy that makes it practically employable in clinical studies. Our model demonstrates a significant improvement in performance, making it a reliable tool for clinical applications.
2. **Addressing Childlike Behavior and Free Play**: Our study is the first to specifically address the nuances of childlike behavior and free play in children with Cerebral Palsy. This aspect is critical, as it aligns the HAR model more closely with real-world scenarios and clinical needs. The listed machine learning models and HAR experiments cited by the reviewer do not appear to incorporate this important dimension, which is essential for developing clinically relevant tools.

We believe that these contributions significantly enhance the technical value of our work and its potential impact in the field of activity monitoring for children with Cerebral Palsy.

In our introduction on page 4, lines 79-86, we have presented existing HAR models for children with CP and explained why our HAR model is still needed. This is further emphasized in our aim on page 5, lines 99-101.

The GitHub repository <https://github.com/ntnu-ai-lab/harth-ml-experiments>, provided by the user, contains well-documented code, except the saved model files. The author should publish the model file in the GitHub repo for the reproducibility of the result. The GitHub repository already contains the code for BILSTM and CNN on the same datasets. Therefore, the authors are requested to include the results of the deep-learning-based experiments (biLSTM, CNN) in the current manuscript to justify the technical contribution and the PLOS ONE journal's reputation. There are already a few existing resources (research papers, HAR datasets, GitHub public repository containing ML, Deep learning and other methods for HAR). There has been no significant improvement in technical contribution from the first submission except for the improvement in writing and clarity of the manuscript.

We appreciate your suggestion to enhance the reproducibility of our results. To this end, we will publish the model file in our GitHub repository (<https://github.com/ntnu-ai-lab/harth-ml-experiments>). We have made changes to our manuscript on page 10, line 211.

Regarding the additional deep-learning-based experiments, we would like to refer to our previous response in not altering our scope and not going beyond our aim of a clinically relevant tool. Thereby we would like to clarify a few points:

1. **GitHub Repository Content**: The existing code in our GitHub repository does not include implementations of biLSTM and CNN models for the specific dataset used in our current study. The code available is for different datasets, and applying these models to our dataset would require extensive additional work, including data preprocessing, hyperparameter tuning, and prolonged training periods.
2. **Scope of Our Study**: As we have emphasized in previous responses, our study's primary goal is to address a specific clinical need using established methods. Expanding the experiments to include deep learning models like biLSTM and CNN would significantly alter the scope of our research and deviate from our original objectives. Our focus has been on creating a practical, clinically relevant tool, rather than exploring a wide array of technical methodologies.
3. **Technical Contribution**: We believe that our contributions lie in the successful application of technical methods to real-world clinical settings, achieving a level of accuracy that makes our model practically employable. Additionally, our work addresses childlike behavior and free play in children with Cerebral Palsy, a critical aspect that has not been incorporated in existing models.

We hope this clarifies our position and the rationale behind our decisions not to do additional experiments. We are committed to making our work as reproducible and transparent as possible.

Some references (published articles, HAR datasets, public GitHub repositories) are provided below:

Existing related papers on the HAR:

1. D. Ravi, C. Wong, B. Lo and G. -Z. Yang, "Deep learning for human activity recognition: A resource efficient implementation on low-power devices," 2016 IEEE 13th International Conference on Wearable and Implantable Body Sensor Networks (BSN), San Francisco, CA, USA, 2016, pp. 71-76, doi: 10.1109/BSN.2016.7516235. keywords: {Machine learning;Feature extraction;Spectrogram;Convolution;Time-frequency analysis;Data mining;Deep Learning;Low-Power Devices;HAR;ActiveMiles},

2. M. Mostafavizadeh, A. R. Sadri and M. Zekri, "Walking pattern classification in children with cerebral palsy: A wavelet network approach," The 16th CSI International Symposium on Artificial Intelligence and Signal Processing (AISP 2012), Shiraz, Iran, 2012, pp. 243-249, doi: 10.1109/AISP.2012.6313752. keywords: {Classification algorithms;Acceleration;Force;Legged locomotion;Entropy;Feature extraction;Accelerometers;Cerebral Palsy;kinetic data;Accelerometer;Pattern Classification;Wavelet Network;Shannon entropy},

3. Csizmadia, G., Liszkai-Peres, K., Ferdinandy, B. et al. Human activity recognition of children with wearable devices using LightGBM machine learning. Sci Rep 12, 5472 (2022). <https://doi.org/10.1038/s41598-022-09521-1>

4. Taborri, J.; Scalona, E.; Palermo, E.; Rossi, S.; Cappa, P. Validation of Inter-Subject Training for Hidden Markov Models Applied to Gait Phase Detection in Children with Cerebral Palsy. Sensors 2015, 15, 24514-24529. <https://doi.org/10.3390/s150924514>

5. J. Kamruzzaman and R. K. Begg, "Support Vector Machines and Other Pattern Recognition Approaches to the Diagnosis of Cerebral Palsy Gait," in IEEE Transactions on Biomedical Engineering, vol. 53, no. 12, pp. 2479-2490, Dec. 2006, doi: 10.1109/TBME.2006.883697.

6. Pengxi Fu, Jianxin Guo, Hongxiang Luo, LightGBM for Human Activity Recognition Using Wearable Sensors. Automation and Machine Learning (2024) Vol. 5: 113-118. DOI: <http://dx.doi.org/10.23977/autml.2024.050114>

7. Malekzadeh, M., Clegg, R., Cavallaro, A., & Haddadi, H. (2021). Dana: Dimension-adaptive neural architecture for multivariate sensor data. Proceedings of the ACM on Interactive, Mobile, Wearable and Ubiquitous Technologies, 5(3), 1-27.

8. Phyo, C. N., Zin, T. T., & Tin, P. (2019). Deep learning for recognizing human activities using motions of skeletal joints. IEEE Transactions on Consumer Electronics, 65(2), 243-252.


HAR datasets:
1. UCI-HAR : <https://archive.ics.uci.edu/dataset/344/heterogeneity+activity+recognition> - containing 30 users performing 6 activities. Accelerometer and gyroscope data were collected by a smartphone worn on the waist

2. UTwente: <https://www.mdpi.com/1424-8220/16/4/426> includes data of 10 users performing 13 activities using accelerometer, gyroscope, and magnetometer data collected from the device on the (right) wrist.

3. MobiAct: <https://www.scitepress.org/papers/2016/57924/57924.pdf>

4. MotionSense: <https://arxiv.org/abs/1802.07802>

Public Github repository on HAR:
1. <https://github.com/mmalekzadeh/motion-sense/tree/master>

2. <https://github.com/mmalekzadeh/dana>

3. <https://github.com/guillaume-chevalier/LSTM-Human-Activity-Recognition>

4. <https://github.com/aqibsaeed/Human-Activity-Recognition-using-CNN>

Reviewer #3: (No Response)

7. PLOS authors have the option to publish the peer review history of their article ([what does this mean?](https://journals.plos.org/plosone/s/editorial-and-peer-review-process#loc-peer-review-history)). If published, this will include your full peer review and any attached files.

If you choose “no”, your identity will remain anonymous but your review may still be made public.

**Do you want your identity to be public for this peer review?** For information about this choice, including consent withdrawal, please see our [Privacy Policy](https://www.plos.org/privacy-policy).

Reviewer #2: **Yes:** Dr. Durgesh Kumar

Reviewer #3: **Yes:** Armel Crétual
